# Quarterly Periscope; or, Spirit of the Public Journals

**Published:** 1822-12-01

**Authors:** 


### Quarterly Periscope

OF

### PRACTICAL MEDICINE;

BEING THE

### Spirit of the Public Journals,

FOREIGN AND DOMESTIC.

*Paucis libris immorari et innutriri oportet, si velis aliquid trahere, quod in animo fideliter hæreat.* SENECA.

Duo vitia vitanda sunt in cognitionis et scientiæ studio. ***** Alterum est vitium, quod quidam nimis magnam operam conferunt in res obscuras atque difficiles, easdemque non necessarias. CICERO.

1. *Series of Metastatic Inflammations.** The subject of Dr. Fallot's paper was a French emigrant, who spent the first twenty years or more of his life in England, and experienced many vicissitudes of fortune. In 1810 he was employed by an English mercantile house as a supercargo to the Isle of France, and to Batavia, where he experienced an attack of cholera morbus, but was cured by calomel in large doses. In 1814 he returned to England—got married—was turned out of his employ—quarrelled with his wife, and separated from her. He then embarked in a merchant vessel for the Mediterranean, where he contracted such an inveterate itch that he came home like a leper. He was cured of this in four months by arsenical medicines—nevertheless a pruriginous affection remained which annoyed him a good deal in sudden changes of weather, and on drinking liquors. In 1817 he travelled to Germany as a commercial agent—had some success, and became rather easy in his circumstances. In the summer of 1818 he became affected with a humid eruption (dartre humide) which occupied the arms and backs of the hands, and which was subdued by a white ointment, the composition of which he did not know. After this event he began to perceive that his disposition was become more sombre, unsociable, and unsettled. He now drank hard, to drown melancholy—and plunged into every kind of excess and debauchery, during which he suffered a severe attack of gonorrhœa. It is not difficult to imagine that this kind of life did not contribute much to tranquillize our patient's mind. In 1820 he began to experience considerable uneasiness in the epigastric region—his digestion became slow and

* Dr. Fallot, Physician at Namur. Journal Complémentaire, August 1822.

distressing—the bowels torpid—his spirits depressed. He now reproached himself for the manner in which he had used his wife and deserted his family. He took measures to be reconciled to them, and succeeded. This procured a temporary calm for his troubled mind. But ennui again assailed him, and it was on the 15th of September, 1821, that he solicited the advice of Dr. Fallot at Namur, whither he had repaired from Aix-la-Chapelle. His complaint, at this time, was a deep-seated and acute pain in the right knee, which he attributed to a twist in getting out of the voiture two days previously.

With the exception of want of sleep, the other functions were not materially disturbed. The part affected was very much swelled, red, and painful, on the slightest movement. The patient informed our author that he had latterly lived free, eating very heartily, and drinking porter, wine, and spirituous liquors. Twenty leeches were applied, a lavement exhibited, and acidulated ptisan ordered for drink. 16. The inflammation but little reduced. Another application of the leeches, and continuance of the regimen. The pain was now removed, and there only remained some sense of numbness and weight in the part, which were dispersed by the 25th, when he called to bid adieu to his doctor. On the evening of the 26th, (having determined to set off to see his family the next morning,) he supped heartily, and drank freely. Scarcely had he lain down in his bed, when he experienced severe pain in the epigastrium, nausea, head-ache. Having drunk some tea and vomited, he felt easier, but on getting into the diligence next morning, he was seized with severe rigor, pains in his joints, and head-ache, succeeded soon by tension and exquisite sensibility of the abdomen, constipation, and thirst. In this state Dr. Fallot again visited the patient. His tongue was now red and dry, the pulse frequent and hard—the features sharp. He saw immediately a case of gastro-enteritis complicated with peritonitis. Twenty-four leeches to the epigastrium, tepid bath —emollient embrocation—lavements—gum water for drink. In the evening the abovementioned symptoms were much mitigated, but a new train of evils had appeared. He was harrassed excessively with ischuria. Sixteen leeches to the pubic region, and the other means continued. 28th. Had a bad night, the urine passing guttatim, and with intolerable sense of scalding (cuisson insupportable.) The urine was red as blood. The gastro-enteritic symptoms being nearly gone, the patient expressed a desire to eat, with which the doctor very properly refused to comply. The patient, however, determined to indulge his appetite; and the doctor took his congée in consequence. On the 30th Dr. F. was again summoned. The urine was again very scanty, the abdomen tense, the region of the bladder prominent, skin hot, tongue dry. The patient had imprudently and vainly been attempting to draw off his own water. Twenty-four leeches to the hypogastrium, anus, and perineum, after which the patient was plunged into a tepid bath, where he had not been more than a quarter of an hour before the urine began to flow abundantly. He was now grown a little wise by experience and conformed to the rules laid down for his guidance. But meeting with some pecuniary disappointments, he was forced to leave his "Auberge," and retire to an obscure lodging, where he gave Dr. F. the history of his former life, of which we have presented the particulars at the beginning of this paper. Dr. F. now saw at the bottom of all the complaints just described, the *repressed* cutaneous affections under which the patient formerly laboured, and proposed an outlet on the surface, in the form of a cautery on the arm. This was complied with on the 17th of October. While waiting for a remittance, and while living in the most frugal manner possible, he enjoyed better health than he had done for many years. On the evening of the 30th of October, (having received his remittances, and being on the eve of departure for his family,) he drank a few glasses of rum grog, and was that night seized again with horrible pain of head, heat about the epigastrium, flatulence, constipation of the bowels, lassitude, despondency. Considering the cerebral affection as only sympathetic of the gastric, Dr. F. merely prescribed diluents and low diet; but he was deceived, for the pains in the head continued after the stomach had become easy, and the bowels free. In a little time, however, the head-ache gave way. The right elbow now became red, swelled, and painful. Blisters did not relieve it; but the burning of three moxas dissipated the inflammation. On the 25th November there was a new attack of gastro-enteritis, complicated this time with hepatitis, the liver being swelled out beyond the false ribs, and the hypochondrium so painful that he could not bear the weight of the bed-clothes. The patient vomited an oily, green, and most acrid bitter matter, the pulse being very quick. Twenty-one leeches to the epigastrium and region of the liver—poppy fomentations to the side. The pain and swelling subsided, and an icteritious suffusion overspread the whole surface of the body. The patient lost appetite, got weaker and thinner, became melancholic, and appeared disgusted with life! When these symptoms had gone to an extent that gave great alarm to Dr. Fallot, lest he had carried his epigastric leeching rather too far, a copious dartrous eruption appeared on the left arm and thigh, the anorexia and general malaise immediately vanishing. He was advised not to apply any thing but warm water to this eruption; but, overcome by the itching, he washed the parts with a preparation of lead. This was on the 5th of December, and on the 8th the symptoms of visceral irritation were kindled up with an intensity greater than ever. The depletive measures being again proposed, the patient positively refused to comply, and swore that he would eat and drink as much as he possibly could, his life being a load of misery to him! He therefore set to forthwith on a *befstek* and a bottle of wine, to the terror and amazement of the doctor. The night was spent in great distress—and next day he passed to the other extreme, and took nothing whatever. On the 11th he received agreeable news from his family, which cheered him up a little; but on the 12th, things took a most alarming turn. About mid-day he was found lying on his bed, his eyes open, and in a complete state of insensibility. Dr. Fallot did not see him till eight o'clock in the evening, when he found him in the following condition :—face slightly tumefied—mouth closed—teeth firmly in contact—eyes fixed and insensible to the light—sense of hearing apparently obliterated—and the olfactory sense in the same condition, for no sternulatories had any effect. Our author endeavoured to rouse the sensibility of the skin and the mucous membrane of the intestines, by mustard cataplasms to the former, and stimulating glisters to the latter, but without success. Towards midnight strong convulsions supervened on this state of insensibility, and threatened immediate destruction. At this critical moment a profuse perspiration broke out over the whole surface of the body, of the most intolerable fetor, and the convulsions gradually subsided. This fetid perspiration continued all next day, and was of such a nauseabund quality that Dr. F. could not get rid of the odour and even taste of it for many days. The patient was now in a state of such extreme debility that his voice was hardly audible. Some wine was administered, and he revived a little. On the evening of the 13th, his lower extremities were observed to be partially covered with large pustular eruptions filled with purulent matter, (" de gros boutons isolés et remplis de pus,) while from the cautery on the left arm flowed an ichorous discharge, having the exact fetid odour of the perspiration abovementioned. He slept some hours during the nights of the 13th and 14th of December—the appetite returned keenly—the spirits got up—the strength improved, and by the 25th December, he was able to set out on his journey to England, where he has since continued in good health.

The foregoing case is, we think, interesting, and affords a good illustration of those mutations and conversions of diseases, or at least of their forms, which so frequently cross our path in practice. A doubt may exist whether the series of visceral irritations and inflammations were owing to repelled cutaneous affections in the beginning. It is to be remembered that after a life of intemperance and debauchery on the Continent, he evinced dyspeptic symptoms, which were succeeded by acute inflammation in one of the lower extremities, most likely of a rheumatic or gouty nature, and it was *after* the dispersion of this local inflammation that the train of internal maladies began to make their appearance. Although therefore, we are well aware of the importance of cutaneous eruptions, and of the danger of repelling them; yet we should be inclined to view the series of phenomena above detailed, as owing, in a great measure, to erratic gout or rheumatism. We leave the facts, however for the consideration of our brethren at large.

---

2. *Cynanche Cellularis.** The case related by Dr. Gregory is, unquestionably, not of common occurrence, especially in the parts here

* Dr. Gregory. Med. and Phys. Journal, No. 284.

affected. The patient was a servant maid, 25 years of age, who was attacked, on the 13th of February, 1821, with feverish symptoms, and pains of the back part of the neck, resembling rheumatism, for which she was bled, and had some opening medicine. The following day, she came under Dr. Gregory's care, evincing considerable fever, attended with great difficulty of swallowing, and swelling, hardness, and some tenderness of the external parts of the throat—chiefly at the junction of the sternum with the clavicles. Nothing particular could be seen on inspection of the fauces. No hoarseness nor difficulty of breathing. During the night, the difficulty of deglutition increased, and the breathing became impeded. Venesection to sixteen ounces, with very trifling relief. Mucus now began to be collected in quantity about the glottis, and was expectorated with great pain. The difficulty of breathing increased to such a degree, that the tongue assumed a blue colour. Blood was twice drawn from the arm, but without more than momentary relief. Leeches and fomentations were resorted to, but the patient died on Monday, the 19th of February, seven days from the invasion of the disease.

On dissection, the cellular membrane beneath the skin of the throat, and around the trachea, as well as that which connects the pharynx and palate to the surrounding parts, was every where in a state of disease—"doubtless the result of inflammatory action." In some places, actual sphacelus had occurred, in others, a state that might be termed imperfect suppuration. The same disorganized condition of the cellular membrane pervaded the whole extent of the anterior mediastinum, even as low as the ensiform cartilage. The mucous membrane of the palate, pharynx, œsophagus, and trachea, was healthy, except that it was covered with a preternaturally abundant secretion of mucus. The lungs and other viscera were sound.

The above disease appears to us, to be an unequivocal specimen of that dangerous species of erysipelas, which attacks the cellular membrane under the skin and between the muscles. In the year 1809, the crew of His Majesty's ship, Royal Oak, suffered severely from this disease, while cruising in the Bay of Biscay. Some lives were lost, and the disease appeared to be contagious, running through a considerable number of the ship's company. It would destroy the whole cellular membrane of a limb in a few days, and when the integuments were slit open, they would fall completely off the muscles, which were left as clean as if they had been carefully dissected. Nothing but free and early incisions through the integuments, so as to allow of the exit of matter and cellular sloughs, saved the life or limb. In several instances it attacked the trunk of the body, and two or three cases proved fatal. The carpenter, Mr. Dalrymple, died in a state of coma. The fever attending the disease was of a typhoid type, and did not bear depletion.

Mr. Thompson, who is now in one of His Majesty's yachts, was surgeon of the Royal Oak at the time, and had a most arduous task to go through. The writer of this article occasionally visited his patients with him.

3. *Variola—Vaccina.** Dr. Forbes, so favourably known to the profession by his translation of Laennec, has witnessed one of those unfortunate epidemics, occasioned by the want of a general adoption of vaccination, and the artificial introduction of variola among unprotected subjects. Dr. F. considers it not only unfair, but decidedly injurious to the cause of vaccination, for medical men to attempt to maintain the same high ground which they formerly assumed, in respect to the almost infallible prevention of small-pox by cow-pox. Yet, "happily, the plain truth is still extremely consolatory;" for every successive year, and every fresh diffusion of the variolous pestilence, tend, more and more, to confirm the belief that the proportion of cases in which vaccination affords perfect security against small-pox, will be extremely great. The same progressive and accumulating experience proves that, "in the small proportion of cases wherein cow-pox fails to prevent variola, it almost invariably, and greatly, mitigates the terrible symptoms of that disease." Our able author thinks, that if, on the promulgation of vaccination, we had been promised *one half* of the benefits which are now proved to result from it, we should never have heard those lamentations, fears, and despondencies, and still less any of that decided preference of small-pox, which are to be found among many members of the community, and even of the profession. The mind is dissatisfied because it has been disappointed—

Jam tenet Italiam, tamen ultrà pergere tendit.

The district which was the scene of the variolous epidemic under review extends along the coast, about 20 miles, and inland about 10 miles, bearing a population of about 30,000 souls. Since the year 1812, variolous inoculation had been almost entirely disused, so that nearly all the children born in the district, since the period abovementioned, had either been vaccinated, or left unprotected *in toto*. A considerable proportion were, unfortunately, in the latter condition, owing to vaccination having been much less practised, especially among the lower classes, than it ought to have been. In 1821, when the inhabitants of Chichester were in great trepidation, the importunities of many persons among the middle and lower orders, to have their children inoculated, were very great; but, to the honour of the profession there, variolous inoculation was uniformly denied—except to such persons as were decidedly exposed to the infection, and whose parents refused the protection of vaccination. Some extra-professional inoculators, however, were at work, and there were nearly 300 cases, casual and inoculated, of small-pox in Chichester. In the vicinity, several extra-professional inoculators spread the disease in all directions, some two or three thousand people having been inoculated!

"A few instances of the failure of vaccination entirely to prevent the attack of variola, were magnified into a total failure of its pro-

* Dr. Forbes. Med. Repos. September, 1822.

tecting powers, while the opinion of perfect security, afforded by the variolous inoculation, was loudly and eagerly proclaimed." In this state of alarm and prejudice, one of the regular practitioners gave way, and his example was followed, as a matter of course and almost of necessity, by his brethren. Accordingly, the surgeons of Emsworth, Havant, and the vicinity, inoculated with great vigour during a period of six or eight weeks—most of these gentlemen, however, representing to the parents and friends of the unprotected, the preferable security of vaccination.

In the above period, the surgeons inoculated more than 1400 persons not previously vaccinated—the whole number, by regular and irregular practitioners, being considerably above 3000. The inoculated small-pox was very mild, the surgeons not having lost more than six or seven cases out of 1450. What was the proportion of deaths in the practice of the itinerants could not, of course, be ascertained—it was evidently, however, very small. There were not more than 130 or 140 cases of casual small-pox among the unprotected. Such was the diffusion of the variolous infection, that it is highly probable that, every individual, who had been vaccinated for many years past, was now exposed to its influence.

"Many striking facts, illustrating the very thorough exposure of the vaccinated, were mentioned to me by almost every surgeon. In a vast number of families, children were inoculated with small-pox, whose parents or elder brothers and sisters had been vaccinated, and who now acted as nurses to their less fortunate juniors. In one family, consisting of eleven, the four elder children had been vaccinated a good many years ago; on the present occasion, the remaining seven were inoculated with small-pox. All the former completely escaped, though they all lived, and some of them slept, together. In one family, consisting of a good many members, all the children were inoculated, except one, to whom the surgeon, on account of a recent burn, refused to give so severe a disease. This child was vaccinated, and resisted the small-pox infection, though living surrounded by, and sleeping with, its pestilential brethren. Hundreds of instances, affording precisely similar results, and many of them equally strong, could be mentioned. In a good many families the remaining children were vaccinated, after the casual small-pox had made its appearance in some of the members, and all escaped (with a single exception,) in whom the vaccine vesicle had time to form. This effect was witnessed in several families, in which death followed the natural small-pox.

"Under all these circumstances of most extensive and most intimate exposure, about eighty cases of small-pox, in persons previously vaccinated, came under the observation of all the surgeons of the district (nineteen in number), during the whole course of the epidemic. All these cases (with a single exception) were completely *modified*, according to the now technical meaning of that word; and, although a few had considerable eruptive fever, and a still smaller number had a considerable eruption of pustules, the disease almost uniformly exhibited the rapid declension characteristic of the secondary affection, and none of the patients were at any time considered to be in danger. As all these subjects exhibited the disease in the *modified* form, it seems to follow, as a necessary consequence, that they had all been vaccinated, and had properly undergone the vaccine process,—as it is only thus we can account for the modified character in any case; it is, however, proper to state, that only a very small proportion (about one-sixth) were known to the surgeons to have undergone the process satisfactorily. In a great majority of these cases of secondary or modified small-pox, the disease was extremely slight, consisting, in some, of a trifling degree of febrile disorder for one or two days, followed by an eruption of a few pustules; and in others, of an equally slight eruption, with scarcely any perceptible fever."

Dr. Forbes has mentioned one exception to the modifying power of vaccination. This was a child twelve years of age, who, after having been casually exposed to the infection of small-pox, was vaccinated by one of the surgeons in Chichester. The arm put on the usual appearances, and the disease went through its regular stages. Three weeks, however, after the period of vaccination, and apparently without being again exposed to variola, the child was seized with the latter disease and died.

Dr. F. received accounts from his medical friends of 680 cases of previously vaccinated individuals subjected by them to variolous inoculation. Of this number only about 30 cases are reported as exhibiting any indication of a constitutional affection from the small-pox virus. In all these cases the resulting disease was completely modified, and with one or two exceptions, extremely mild.

So much for the protection of vaccination. There were seen, during this epidemic, nineteen well authenticated cases of second attacks of small-pox—in most instances after inoculation. Some of these secondary attacks nearly proved fatal.

Upon the whole, the effect of this epidemic is, our author thinks, "a diminution of prejudice against vaccination, and an increased confidence in its preventive powers," among the common people. This is a great advantage, and we have only to thank our able author for his clear, perspicuous, and well written communication on so important a subject.

---

4. *Puerperal Fever.** Mr. Moir relates a case of puerperal fever, and accompanies it with some observations which we deem to be of an extraordinary nature. We shall first give the outline of the case itself. A lady, in the 28th year of her age, was delivered (breech presentation) at three o'clock, on the 19th June, 1822, and seemed to be doing well through the night. Next morning, however, her pulse was feeble, and she had a fatigued appear-

* Mr. Moir. Ed. Journal, No. 73.

ance. The uterus felt harder than usual, and there was some abdominal tenderness, apparently from flatus. The lochial discharge was natural. At 2, P. M. she had a rigor, accompanied with much pain and tumefaction of the abdomen, and a dull heavy pain in the forehead. Her countenance now appeared pale, and expressive of great anxiety—her breathing was short and frequent, but not laborious—skin hot—pulse 120, weak and easily compressed—abdomen greatly swelled, and tender to pressure—some sickness and faintness; but "the chief seat of uneasiness was in the uterus and right iliac region." When perfectly quiescent, the pain could be tolerated without complaining; but the least movement of the body, or pressure on the part, rendered it excruciating. An ordinary glyster brought away "two full costive and fetid evacuations." Even this produced such a degree of exhaustion that both wine and æthereal cordials were necessary. In the evening, although some relief had followed the enema, all things had got worse again—the abdominal pain had not decreased—the head-ache was greater—the pulse 130, small, and fluttering. The cordial medicine was continued, and a drachm of the tincture of hyoscyamus to be taken immediately.

On the 21st, the symptoms were aggravated—pulse 140, small and equal—breathing short and hurried—cheeks a little flushed, while a death-like paleness overspread the rest of the face—head-ache unabated—skin hot—abdomen swollen. Three stools had been procured by an enema—lochial discharge natural. A dose of castor oil; and the cordial mixture to be continued. At four in the afternoon manifest relief had resulted from three evacuations by the castor oil. Much flatus had come away; but the state of the pulse and other symptoms was not altered—the debility was increased. Two glasses of wine, with panada, were ordered in three or four hours. In the evening the alarming symptoms had increased, though the abdominal swelling had diminished—pulse 150, and remarkably feeble—cessation of the mammary secretion—lochial discharge continued, though pale. The cordial mixture was ordered to be repeated; and an anodyne draught with ʒj. tinct. hyoscy. and ♏. xx. tinct. opii, at bed time. 22d. All the symptoms more favourable—had slept and perspired in the night—pain in the head and abdomen greatly relieved—swelling of the belly much diminished—pulse 130 and stronger—tongue clearing at the edges. A dose of castor oil—some wine. From this time her convalescence went on regularly.

Now on this case we shall take the liberty of remarking that, so far from considering the above-described symptoms as unequivocally marking it "the malignant puerperal fever described by Hulme, Doublet, and other authors," we unhesitatingly pronounce it to be a very exquisite specimen of what Dr. Marshall Hall has admirably described, though somewhat vaguely denominated, "a serious morbid affection chiefly occurring after delivery, &c. from various sources of irritation and exhaustion." The principal source of this irritation Dr. Hall has traced to *a disordered and loaded state of the bowels.* In short, if any one will turn to Dr. Hall's little work, or to our review of it in the first volume of this series, p. 195, he will see the disease, denominated by Mr. Moir "malignant puerperal fever," fully delineated. We cannot therefore but deprecate the sweeping dogma that would amalgamate this affection with puerperal fever, or puerperal peritonitis, and proscribe the lancet altogether in a complaint where it is our principal resource. As for the peculiar opinion of Mr. Moir's old preceptor, Dr. Hamilton, that in real puerperal fever the lochia continue, and in common hysteritis or peritonitis they are suppressed, we can only say that the profession at large recognize no such distinction, and only consider it a little hobby of the doctor's which he may fairly be allowed to ride about in the class-room for his own health, and the edification of his pupils. Mr. Moir's acquaintance with the variety of forms which diseases assume may be estimated by the following dogmatical assertion. "In all of the cases in which these gentlemen (Drs. Gordon, Armstrong, Hey, &c.) succeeded, the pulse beat from 120 to 140, and the lochial discharge was suppressed at the very commencement of the disease—symptoms which, I do not hesitate to affirm, *no intelligent practitioner of respectability* ever met with in a real case of malignant puerperal fever." Now one of the two symptoms here denied to malignant puerperal fever, viz. pulse from 120 to 140, was actually present in Mr. Moir's own case; so that by his own dogma Mr. Moir can only pretend to be *half* of an intelligent and respectable practitioner. Mr. Moir says that the descriptions given by the above-mentioned gentlemen are contradicted by the detail of the individual cases. They may return the compliment, for Mr. Moir's dogma is unequivocally contradicted by the case which he has brought forward to support it.

P. S. The following case has just passed under our notice. Mrs. Moore, 24 years of age, was delivered of her first child on Saturday, the 19th of October, after only two hours of labour. The placenta came away spontaneously. On Sunday she was seized with a severe and long-continued rigor, succeeded by great reaction, acute pain and tenderness of the abdomen, especially above the groins, with a pulse of 120. Mr. Bagster saw her and bled her to nearly 30 ounces, when tendency to syncope took place. The blood was deeply buffed. A cathartic was exhibited, which operated. On Monday, Mr. Bagster found the pulse 130, and all the inflammatory symptoms again increased. She was again bled nearly to syncope. On Tuesday we saw her with Mr. Bagster. The pulse was 146—the abdomen exquisitely sensible, and a little tumefied—the skin rather hot—the face flushed—constant anxiety and tossing about in the bed—the lochia nearly suppressed this day, and not before—no afflux of milk to the breasts—delirium during the preceding night, but now calm and collected. The pulse was very small, with the above-mentioned rapidity. We expressed to Mr. B.

our opinion that inflammatory action was still going on, and that although there was little chance of recovery, yet depletion was still the only means that afforded that chance. Thirty leeches to the abdomen, and after they came off and bled a few hours, the whole belly nearly was covered with a blister. Three grains of calomel and a quarter of a grain of opium were ordered every four hours. She slept two or three hours on Tuesday night, and had two stools, but of a dark watery kind, in the morning. On Wednesday we found her much relieved in respect to pain and tenderness of the abdomen, but the pulse was 160, and still smaller than ever. We then considered that effusion had taken place. She had no sickness at stomach till Thursday, and she died that night. Mr. B. opened her, and Dr. Cowie was present with us. Full two pints of sero-purulent effusion mixed with flakes of coagulable lymph were found in the abdomen. The peritoneum, in many places on the small intestines, was inflamed and nearly gangrened—both ovaries were in a state of incipient gangrene—and the right one was surrounded with a quantity of thick puruloid matter. The uterus, when slit open, presented marks of inflammation on its internal surface.

Now what will Mr. Moir, or his oracle Dr. Hamilton, call the above disease? Had no dissection taken place, it would have been puerperal fever, and the patient, of course, was killed by depletion:—but dissection having amply justified the means that were used—then, we suppose, it will be denied that it was *real* puerperal fever!

---

5. *Tar Vapour.** Dr. James Forbes, Deputy Inspector of Military Hospitals, has given the inhalation of tar vapour, as recommended by Sir A. Crichton, a trial in pulmonary affections, the result of which corresponds with our own experience in this remedy. It is, as Dr. Forbes observes, a difficult thing sometimes, to distinguish between chronic affections of the mucous membrane, and tubercular ulceration in the parenchymatous structure of the lungs. In the latter state tar vapour, like all other remedies, fails. But it does more—it aggravates the complaint, as we ourselves have witnessed. In chronic catarrhal complaints, however, it often is of service. We can scarcely agree with our author in his pathology of chronic catarrh. He considers it to be a disease succeeding inflammatory action of the bronchia, "but which is itself unattended with *any lesion or inflammation;* but, on the contrary, has for its cause a morbid relaxation of the lining membrane of these tubes." This "morbid relaxation" must surely be some kind of "lesion," even if it be not of an inflammatory nature. But we are far from

* Dr Forbes. Med. and Phys. Journal, 284.

believing that this morbid relaxation, and this "preternaturally increased secretion" of the mucous membrane of the lungs is unconnected with chronic inflammation. On the contrary, we think they are, if not one and the same, at least twin brothers, or probably as cause and effect with each other. Dr. Forbes illustrates his pathology by the state of the urethra in gleet; but really we think gleet exhibits an unequivocal example of chronic inflammation in the mucous membrane of the urethra, as a mucous diarrhœa does that of the lining membrane of the intestines. Be this as it may, we shall give the result of our author's experience with tar vapour.

| Diseases. | Total. | Cured. | Improved. | No Effect. | Bad Effect. |
|---|---|---|---|---|---|
| Phth. Pulm. | 19. | none. | none. | 8. | 11. |
| Catar. Chron. | 32. | 8. | 6. | 18. | none. |

6. *Apparent Pregnancy.** On the 6th June, 1820, Dr. Dewees was requested to see a young lady who had been suddenly taken ill with severe pain in the uterine region, and almost instantly afterwards with delirium and mental disturbance. She complained much of her head, palpitation of the heart, and of occasional sense of suffocation. She had frequent lucid intervals from the delirium. She had not menstruated for more than a year—her belly was much swelled —her breasts enlarged—she had morning sickness—and "all the usual signs of pregnancy." Dr. D. on examining the abdomen, felt a circumscribed tumour within it, which, he was very certain, was an enlarged uterus, and he thought he distinctly perceived the motion of a fœtus. As there were febrile symptoms, bleeding and other antiphlogistic measures were ordered, which removed the symptoms before described, and she remained well for a fortnight. At the expiration of this period they again returned, precisely as before, and were again dissipated by the same remedies. The symptoms returned twice more, and twice more they were removed. Mean time, the abdomen continued to enlarge, and the feet and ankles to swell. On careful examination, it was found that there was a fluid in the abdomen, evinced by evident fluctuation. As the young lady's character was beyond reproach, and as there was no perceptible increase of the *uterine* tumour during the last two months, Dr. D. began to conclude that he had ascites instead of pregnancy to deal with. He therefore prescribed the volatile tincture of guaiacum, in doses of a tea-spoonful thrice a day in a wine-glass full of milk. After taking this medicine for a few days, it purged her very briskly, and made her discharge very large quantities of urine. Some few drops of laudanum being added, she was enabled to persevere in its use for three weeks, without further inconvenience, at

* Dr. Dewees. Philadelphia Journal of the Medical and Physical Sciences, No. VII.

the end of which time no vestige of water was discoverable in the abdomen, " but a serous discharge was observed from the vagina, which was soon followed by a sudden gush of fluid blood, to the amount of about three pints, which soon abated in quantity, and at the end of a week, entirely ceased." She menstruated at regular periods after this, and continued to enjoy good health. We leave our obstetric readers to form their opinions respecting the above case. In a moral point of view it is interesting, as it shows how easily a derangement in the uterine economy may lead to the most cruel and unjust suspicions.

---

7. *Dropsy of the Kidney.** A medical practitioner, ætat. 25 years, had enjoyed good health till the age of 15, when he suffered an attack of nephritis in the left kidney, from which he gradually though slowly recovered—always experiencing pain and inflammatory symptoms after much fatigue or exposure to severe cold; but giving way to gentle purgatives and low diet. In the beginning of February, 1821, he had a very severe attack, reaching in a few days to an alarming and dubious height. In addition to hardness, there was a sense of crepitation in the seat of the pain. He was bled freely, and gradually but partially recovered in six weeks. He had repeated attacks the succeeding summer, but they gave way to evacuations and low diet. After getting stouter than usual, he attempted to ride on horseback. The trotting motion gave him great agony. On the 8th September his final attack commenced, and the symptoms were similar in kind, though intenser in degree, than those which he had been accustomed to experience previously. The principal symptom was a fixed, knawing pain extending from the spine to the umbilical region, increased by pressure, and by motion of the trunk. The contact of air gave him great distress. His urine was small in quantity, and once or twice slightly tinged with blood —it never was milky or purulent. The evening previous to death he complained of inability to make water. His bowels were generally costive, and his pains were aggravated by constipation. Relief, though temporary, always followed alvine evacuations. The pulse at the commencement of the attack was 84, and gradually rose to 120. Bleeding always gave relief for a time. A day or two previous to his decease, what he passed was not fœculent, but gelatinous matter, and was accompanied with most excruciating pain. A strong idea prevailed in his own mind that the complaint was " spasmodic enteritis," a disease of which we confess we cannot form any clear idea. He never had thirst or rigors—his skin was generally moist and bedewed with perspiration. He never experienced much emaciation, even to the last.

---

Dr. Howison.—Ed. Journal, No. 73. Dr. James Johnson.—Med. Chir. Journal, (for July, 1816.)

The body was examined in presence of Drs. Duncan, Alison, and several other medical gentlemen. On opening the abdomen every thing appeared natural at first sight. The liver, on being pulled down, had a blanched appearance, and was very destitute of blood. In tracing the alimentary canal from the stomach downwards, nothing unusual appeared until they came to the sigmoid flexure of the colon, which was considerably thrown forwards from its natural position; and on drawing it out, a large oblong flat tumour, occupying the whole lumbar region and part of the ilium, lying behind the peritoneum, and evidently containing a fluid, presented itself. This tumour extended upwards towards the true ribs, and downwards towards the pelvis—backwards towards the spine; and forwards to near the umbilicus. It measured about a foot in length, and nine inches in breadth, being of a kidney shape. After some labour it was completely insulated from the surrounding parts, and when removed it was found to include the kidney entirely within it. They tied and divided the renal vessels, and the whole being removed it was laid open by a longitudinal incision, when about three pints of fluid escaped. "The tumour was now found to be formed entirely of the dilated kidney, the cortical and medullary part of which had disappeared, except a few small portions, leaving nothing but a cavernous cyst, consisting of the proper external membrane of the kidney, and its internal membrane much thickened. It was divided into three large irregular cells, freely communicating with the dilated pelvis, into the apex of which the ureter, of its natural size, opened." "The fluid did not possess the smell, taste, or any of the peculiar sensible qualities of urine, but was of a *whitish colour*, like pus diluted with serum." On emptying the kidney there was found a very small calculus, which, on being tried, exactly filled the orifice of the ureter.

We shall now proceed to state the outlines of a case almost exactly resembling the above, in all essential particulars, but still more remarkable in many respects. It is very fully detailed by the editor of this Review, in the 2d volume of the Monthly Medico-Chirurgical Journal, for July, 1816. The patient was a poor woman, who came under Dr. Johnson's care, on the 6th May, 1816, being then in the 8th month of pregnancy. She complained of violent pain in the right side of the abdomen, "extending from the umbilicus round to the lumbar vertebræ, and from the floating ribs to the groin." She had much pyrexia, with scanty, high-coloured urine. "On examining the abdomen, it appeared unusually large; but, what was more singular, a distinct *sulcus* was felt, running from the scrobiculus cordis to the pubes, on each side of which line the abdominal tumour rounded out, as if there were two impregnated wombs instead of one. Both sides, too, presented nearly the same kind of surface, except that the right felt rather more uniform and elastic than the left. Pressure on every part of the right side gave great pain quite round to the spine."

But an imperfect history of the complaint could be obtained. She had lately been landed from a small ship of war, and stated that, for two years at least, she had felt more or less pain; it was only since the period of quickening, however, in her present pregnancy, that it had become so very distressing. During the last three months it had been unremitting. She said there was no swelling of the abdomen before pregnancy; but that, in the latter months of utero-gestation, on a former occasion, she had felt more than usual pain of the right side, Her bowels had always been regular, but as to her urine, it was different—it was always either scanty and high-coloured, or "plentiful and white like milk." When in the latter condition she was comparatively easy—when scanty she was in great pain. The miserable woman attributed her present ailment "to a practice which, during the suckling of a former child, she had been forced to adopt, in consequence of being at sea with her husband—namely, that of drying all her child's linen and napkins, by wringing them out hard, and then placing them next her skin (of the abdomen) to dry."*

Between the 6th and 10th of May, she was bled, took aperients, had blisters on the abdomen, and was taking diuretics. On the 10th, she was in labor, and each parturient paroxysm produced great agony. On the morning of the 11th she was delivered of a living child. She now expressed herself as perfectly free from pain.

"On examining the patient (says Dr. J.) this day, I was surprised to find, that the right side of the abdomen was little diminished in size; but, on the contrary, from the shrinking of the left side, a large tumour appeared, projecting at least four inches above the general level of the belly, and seven or eight inches in diameter. It was turgid, and perfectly even all over its surface; on tapping it gently, a fluctuation was evident, and pressure on it occasioned pain."*

No lochia succeeded delivery, and the original pain soon returned, though not in so violent a degree as before delivery. But fever, colliquative diarrhœa, and constant micturition, harrassed the patient. At this time she was seen by Drs. Lara, Gray, Seeds, and several medical gentlemen, and the general opinion prevailed, that the disease was *ovarian dropsy*. The propriety of puncturing the tumour was discussed, but the operation was not deemed adviseable.

"Early (says Dr. Johnson) in the morning of the 20th of May, the nurse was surprised to find that the patient's bed had been completely drenched, during the preceding night, by a milky fluid, which had spread itself over the floor of the room, and descended, in considerable quantity, into a chamber underneath.—From what source the fluid issued the nurse could not tell, and the patient had that night slept so well, that the passage was involuntary; at least, she was unconscious of the circumstance. She asserted, however, that it was the same kind of milky urine which she used formerly to

---

* Med. Chir. Journ. vol. ii. p. 4.

make occasionally. It certainly had very little of the urinous smell, and I did not feel disposed to taste it. On pulling down the bed-clothes, the tumour was gone! Manual examination, however, discovered some fulness and fluctuation on that side, though the rise was not very observable above the general level of the abdomen. The pain was also abated, and whitish urine continued to be made in considerable quantities. Notwithstanding this sudden change, the fever, the rapid emaciation, the entire prostration of strength, the sinking countenance, the cold sweats, and colliquative diarrhœa, proclaimed themselves the harbingers of death. Nature, however, struggled four days with these symptoms, during which time a great deal of the same white water was passed involuntarily, as were also the stools. Early in the morning of the 25th of May she expired, the abdomen appearing as flaccid and reduced as though no previous tumour had existed." 5.

It was with the greatest difficulty, and not without bribery, that Dr. Johnson obtained permission to open the body, and that under some galling restrictions. Dr. J. examined the abdomen, eight hours after death, in presence of Dr. Thomas Seeds, and one assistant. The minutes of the dissection we shall give as published by Dr. Johnson in the volume alluded to.

"An incision being made from the sternum to the umbilicus, and from the umbilicus to the spine of each ilium, the triangular flap of integuments was everted over the pubes, when the CAPUT COLI first presented itself, much distended, and *apparently* with an immense pouch attached to it and the ascending portion of the colon, which pouch filled up the hollow of the ilium, from the liver to near the groin, and from beneath the anterior parietes of the abdomen back to the spine. On looking down into the cavity of the pelvis, when cleared of the small intestines, the bladder was seen moderately distended; the uterus sufficiently contracted for the length of time after parturition; the ovaria and Fallopian tubes on each side, perfectly distinct, natural, and healthy!

"Returning to the colon, on a close examination, and before attempting to remove the parts *ex situ*, I thought I could perceive something like a sulcus between it and the pouch; and, by a careful and delicate dissection, I separated entirely the caput, and ascending portion of the colon from the bag to which they were so intimately attached, as to appear identified with it. The cyst was next to be detached from the concave surface of the liver, from the hollow of the ilium, and from the posterior part of the peritoneal lining of all the right side of the abdomen round as far as the spine. To these various parts it was most firmly agglutinated, but without a single trace of *recent* inflammation.

"In raising the lower portion of the bag from the hollow of the ilium, a large vessel was exposed, lying in the direction of the psoas muscle, which, at first sight, I took for the external iliac artery; but on clearing it a little from the surrounding cellular substance, I was soon undeceived by coming to the iliac artery with its accompanying vein, and also by observing that the vessel itself was tortuous, and unequal in calibre. In some places it was quite as large as the iliac artery, in others smaller. I first carefully traced it upwards, for a considerable way, and ultimately found it enter the body of the sac:—I next traced it downwards, into the cavity of the pelvis; under the Fallopian tube; and into the bladder. Till this moment, the nature of the disease was doubtful; it was now evident, that the vessel so traced was the ureter, while the immense bag, from which it descended, was the capsule of the kidney distended to a hitherto unexampled magnitude!

" The ureter, which was very turgid, could be emptied of its contents by pressing the fluid downwards into the bladder, but immediately filled again from the sac. A ligature was passed round it near its entrance into the capsule, and, the tube being divided below that, there gushed out nearly two ounces of the same kind of whitish liquid which passed latterly *per urethram*. We now proceeded to detach the capsule from its various attachments to the liver, small intestines, psoas muscle, &c. &c. but found this so tedious and difficult an operation, that the patience of the female spectators became exhausted, and we were forced to hasten the investigation as much as possible, by examining the interior of the bag *in situ*. I had so far separated the capsule, however, that I could pass my hand in all directions round it, except where it adhered to the concave surface of the liver, and where the emulgent vessels entered the bag. I was particularly careful to examine whether the kidney or any part of it remained uninclosed in the cyst; but there was no vestige of the kind. On making an incision into the cyst, six or seven inches in length, we found in its cavity about three pints of the same fluid as came from the ureter, which being sponged out, we had an opportunity of viewing the internal surface of the bag. Its parieties varied a great deal in thickness; from that of a penny piece to that of a shilling. The whole internal surface, however, was highly vascular, and thickly studded with a kind of mammillary, or papillary bodies, varying in size from that of a pin's head, or less, to that of a very small pea. Several thin laminæ, or semicircular septa projected from different parts of the walls of the cyst; but the largest of them never went half across its diameter, so that the freest communication existed throughout the whole cavity; in fact, it was but one cyst, for a large sponge went freely to the bottom of it, and cleared it entirely of its contents.

" The bladder was now punctured at the fundus, and nearly a pint of the same kind of fluid as the cyst and ureter contained, flowed out into the pelvic cavity.

" It is difficult to calculate the quantity which this cyst may have contained when in its maximum of fulness, immediately after parturition; but from the flaccid state in which it now appeared; from the immense flow of water which took place during its subsidence; and from its great extent and projection externally, before the discharge, we are probably under-rating the quantum much, when we say, that at one time it held five or six quarts of fluid."*

Dr. Johnson was at a loss to give the disease a name, and proposed that of "*hydro-renal distention*," on the same grounds as we use the terms hydro-thorax, or hydro-pericardium. This disease differs materially from hydatids of the kidney, as described by our illustrious pathologist Dr. Baillie. Dr. B. relates the case of a soldier, whose right kidney "was converted into a bag capable of containing at least three pints of fluid, and only a very small part of the kidney, at the lower end, retained its natural structure. The bag was of considerable thickness; was obscurely laminated, and had a *cartilaginous hardness upon its inner surface. It was full of hydatids.*"†

In Dr. Howison's and Dr. Johnson's cases, especially the latter, the inner membrane was highly vascular, and, without doubt, a secreting surface. "It appears, in fact," says Dr. Johnson, "that, from some cause or other, the pelvis of the kidney had become distended to the size of the inner surface of the cyst, while the glandular substance had either become entirely absorbed, or was expanded between the pelvic and capsular tissues, forming probably the papillary bodies above described, and still retaining the secretory function."

An interesting question remains, to wit, what is the cause of the disease? We imagine that it is obstruction in the ureter. In Dr. Howison's case we see there was a small calculus exactly fitting the orifice of the ureter, and in Dr. Johnson's, although circumstances did not permit him to examine the ureter with sufficient care, yet it is manifest, from the symptoms during life, that the channel from the kidney to the bladder was occasionally obstructed. There is nothing in Morgagni that bears directly on this subject, unless it be the following passage:—"In the *acta eruditorum* an observation is extant made by Groenvett, on a *calculous* girl, whose ureters resembled one of the small intestines, by their capacity being enlarged. And Mauchartus saw the same canals, in an old man afflicted with strangury, inflated like the *intestinum ilium from urine like butter-milk;* at the same time that the *kidneys were very large and unequal,* and had their *pelvis distended to the magnitude of an egg.*"‡

But the following case, in the sepulchretum of Bonetus, affords very presumptive proof, at least, of the etiology of the renal distention under consideration.

"Ante sex circiter septimanas accessit me chirurgus, indicans monstrosum fœtum pridie natum esse. Cum corpusculum detegerem, vidi ingentem tumorem in abdominis regione, sub hypochondrio sinistro, et facta dissecandi copia, adsumpsit mihi comitem Clariss. Dom. Borrichium, &c. Aperto abdomine cum magna cir-

---

* Med. Chir. Journal, p. 7, 8. † Mordid Anatomy, p. 279.

‡ De Causis et Sedibus, Ep. 40, Art. 24.

cumspectione, invenimus hepar mole sua naturalem quantitatem non excedere, ut nec ventriculum nec lienem. ***** Nam venis magnis per superficiem sparsis preditus erat tumor, et in ejus parte superiore, aliquid rubicundi instar placentæ uterinæ apparebat. **** Aperto tumore invenimus eum repletum fuisse copia seri ingenti; tandemque deprehendimus *renem dextrum in ejusmodi molem excrevisse, et tumorem illum efformasse*, qui tamen ren et a figura sua naturali, et a substantia plurimum discrepabat, cum crassissimæ membranæ erat admodum similis, *ureter quoque dexter plane erat impervius.*"*

Here then we have a case of *congenital* renal distention, with an *impervious* ureter on the same side:—and that these stood in the relation of cause and effect to each other, there can be no reasonable doubt. We think that the same explanation may be extended to those cases that are recorded by the gentlemen abovementioned. We cannot, therefore, agree with Dr. Howison in his pathology of the case detailed by him. He never seems to suspect obstruction of the ureter as the cause of the disease; and, strangely enough, supposes, that the inflammation which "existed in the interior of the kidney arose from the *ulceration* there going on." On the contrary, we believe that the inflammation arose from the distention caused by the obstructed ureter, and that the ulceration was the *consequence*, not the *cause*, of the inflammation.

From this view of the etiology and pathology, it is obvious, that puncturing the tumours would not have afforded any effectual relief, as it would not have removed the cause of the disease.

---

8. *Singular Disease of the Nervous System.*† "M. ——, aged thirty-six years, of an agreeable figure, cultivated mind, but of great nervous susceptibility, led a gay life prior to his marriage, which took place about six years ago. At that time he experienced some crosses in business, and was afterwards severely afflicted, in consequence of a mental derangement which attacked his wife, at the time of her first accouchement. He never left her during the whole of the disease, but accompanied her in a journey, and was thus witness, for nearly a year, of the incoherencies and convulsive affections of a being for whom he had the most tender attachment. The complete cure of Madame —— put an end to the moral torture which her husband experienced; but, instead of giving way to joy, which such a fortunate event might naturally occasion, he remained dull and silent, and gradually shewed every symptom of real melancholy—believing himself inevitably ruined, and feeling persuaded that he was the object of animadversion by the laws, of the searches of the police,

---

* De Hypochondriorum Tumore, Lib. III. Sect. XVII. Obs. XXII.

† M. Magendie. Journal de Physiologie, April, 1822.

and of public raillery. His mind was perfectly correct upon every other subject. He was recommended to travel, to take the waters, and to undergo different modes of treatment, but without success.

"Things were in this condition, when, in the month of September last, he was seized with a degree of stiffness in the right leg and thigh, to such an extent as to cause him to limp. A few days afterwards, a similar stiffness attacked the opposite thigh and leg; and he subsequently lost all power of volition over his movements. The limbs were, however, far from being paralyzed, but they were, in some measure, isolated for whole hours: he was then obliged to execute the most irregular movements, to assume the most whimsical attitudes, and to make the most extraordinary contortions. It is impossible for language to paint the multiplicity and the strangeness of his movements and positions. If he had lived in times of ignorance, he would, doubtless, have passed for one possessed of a devil; for his contortions were so far removed from the movements proper to man, that they might easily have been regarded as diabolic. It was worthy of remark, that, in the midst of these contortions into which his thin, supple body was thrown, sometimes forwards, sometimes behind, or to one side, like certain vaulters, he never lost his equilibrium, and that, in the great number of attitudes and singular movements which he executed during several months, he never happened so fall. In certain cases, his movements re-entered into the class of ordinary movements; thus, without his will participating in the least in the world, he would get up and walk rapidly, until he met some solid body which obstructed his passage; sometimes he started backwards with the same promptitude, and was only stopped by a similar cause. He was often observed to resume the use of certain movements, without being able in any manner to direct the others: thus, his arms and hands frequently obeyed his will; and, more frequently still, the muscles of the face and of speech. It was sometimes possible for him to recoil, when his progress in advance was interdicted, and he then made use of this retrograde movement, for the purpose of directing himself towards the objects which he was anxious to reach.

"Finally: these movements, which might be called *automatical*, never continued a whole day: he had tolerably long, quiet intervals between the paroxysms, and his nights were always tranquil.

"Although the contractions were very violent, so as to produce copious perspiration, when they had ceased, he experienced no feeling of fatigue, proportionate to the intenseness of the efforts which he had made—as if the intellectual exertion which we make use of, in order to excite our movements, is that which becomes the most fatigued in us.

"Reckoning from the day on which the movements showed themselves, there was a slight amelioration in his moral condition.

"The physicians of the province in which he dwelt made use of several means against this singular disease. Amongst others, baths, leeches, antispasmodics, &c. were useless. They then decided upon sending him to Paris, where he arrived in the month of December last, and put himself under my care.

"I employed the first days in studying his condition, which I could not class under any known disease. It was not a catalepsy, for the movements were frequently rapid and multiplied; there were no convulsions, for the contractions had a certain *ensemble*, and a sort of regularity in the disorder of the movements; and it was not the dance of St. Guy, for in that there is an agitation, a friskiness, and a versatility in the contractions, which were not perceptible in this patient.*

"What was to be done in such a case? and how much the emptiness of medical theories is felt upon such occasions!

"Many remedies had been already employed, and it was necessary to try others.

"Having no particular motive, and seeing, moreover, no inconvenience from it, I decided upon administering the sulphate of quinine, which was given in the dose of two grains a-day, in a small potion.

"From the second day an appearance of amendment was perceptible; on the third it was evident, and, on the sixth, all the *automatical* phenomena had disappeared; and M. ——, to his great satisfaction, regained the supreme direction of his locomotive system.

"From this moment (about four months from the present time) he has had several slight relapses, always produced by strong moral emotions, such as the death of his sister and father-in-law. At the first time, I considered it proper to have recourse to the curative means which I had employed with so much success, and I obtained from it a speedy cessation of the symptoms.

"In the last month, there have been some involuntary movements, which continued for some hours, and disappeared of themselves.

"A final remark, which I should not forget, is, that the influence of volition over the movements was gradually restored: for example, during several weeks he was unable to run, and, consequently, felt obliged to be contented with walking, and even with this to a certain limit, both as regarded the extent of his steps and their speed."

---

9. *Stethoscope.*† M. Kergaradec has lately published a small memoir upon the Application of Auscultation to the Study of Pregnancy. By means of the *stethoscope* or *pectoriloque* of Laennec, he is of opinion, that the pulsations of the heart of the fœtus may be distinguished from those of the arteries of the mother; and that, by an attention to the different sounds communicated to the ear, we may determine, in doubtful cases, whether the fœtus in utero is alive or dead. MM. Kergaradec and Laennec are both of opinion, that the

---

* Both the phenomena and the treatment convince us, that the disease was nothing else than chorea, of which there are many instances on record, where the movements were quite as singular as in the present case.—*Ed.*

† M. Kergaradec. Magendie's Journal.

*stethoscope* and the immediate application of the ear, are attended with the same advantages; but M. Fodera considers, that although, for the purposes of delicacy, the *stethoscope* may be sometimes preferred, yet that we may frequently be able to detect diseases by the immediate application of the ear, which we are not able to do with the *stethoscope;* and he consequently gives the preference to auscultation performed in that manner, in all cases where the *stethoscope* may be considered necessary.

10. *Uterine Hæmorrhage.** Although it be a general truth, that uterine contraction secures from uterine hæmorrhage—and that the uterus is contracted when it feels small, round, and firm; yet the attentive practitioner is occasionally struck by the disproportion that exists between the want of contraction and the degree of hæmorrhage—a bulky uterus being often free from discharge—and there being, sometimes, a profuse flow when the uterus is small and contracted.

"Nay, further, I have witnessed a profuse hæmorrhage though the uterus had contracted in the degree which commonly indicates security; and I have ventured to do what is seldom justifiable, separate the placenta before the uterus had contracted, without more hæmorrhage than after a common labour. What is this circumstance which has so great an influence that its presence can cause a moderately contracted uterus to bleed profusely, and its absence can cause an uncontracted uterus to bleed scarcely at all?"

Experience has taught our author that, there are two circumstances in which a hæmorrhage may prove alarming, though the uterus be contracted.

1st. The effects of uterine hæmorrhage are comparative in different constitutions. If some people, who are prone to syncope, lose rather more blood than usual, it will affect the constitution as much as a profuse hæmorrhage will others—this trifling excess not being indicated by any thing unusual in the size of the uterus.

2dly. After delivery, in general, uterine contraction prevents hæmorrhage, by closing the mouths of the vessels so as to resist the *ordinary* momentum of the circulation. But if this momentum be *extraordinarily* great, it is reasonable to suppose, that it might overcome the common occlusion of the vessels even in a contracted uterus.

The two positions above stated, are elucidated by cases. The following is illustrative of the first position. Dr. G. delivered a lady of her first child, the labour being short and easy. About ten minutes, *post partum*, the uterus felt round, firm, and of the usual size. In this state of things, she suddenly exclaimed that she was going to

* An Account of some Circumstances in which a Uterine Hæmorrhage may occur sufficient to produce alarming Symptoms, though the Uterus feels contracted in the ordinary Degree. By Robert Gooch, M. D.—*Med.-Chir. Transactions, Vol.* XII. *Part* 1.

faint, (being accustomed to it,) and, in a minute afterwards, she did faint. For a long time, she continued alternately reviving and sinking—"a state which the most experienced cannot watch without painful anxiety." Dr. G. does not give the sequel, but we conclude, of course, that recovery took place.

In some constitutions the syncope is the accidental effect of an exhausting labour, and is accompanied by that powerless and agitated state of the vital functions, termed "nervous irritation"—cases in which the best cordial is an opiate. We shall insert the following case in the words of our author.

"I was attending a lady, thirty-six years old, in her first labour. Of her mother and three daughters, all but one, have, in their first labours, been delivered with the forceps. She had a severe and protracted labour, and no longer feeling justified in postponing the delivery, I applied the forceps. The external discharge was not greater than usual; the uterus, after the removal of the placenta, felt rather, but not very, large. She had not been tranquil since the extraction of the child, but about twenty minutes afterwards her appearance alarmed me greatly. Her heart beat with indescribable rapidity, her pulse was countless, she breathed so quick and short that, to use her own expression, if it became quicker and shorter she should not breathe at all, and she felt as if she was dying.

"I had two duties to perform; one, to take care that the symptoms were not kept up, nor caused by internal hæmorrhage; the other, to administer what I thought most likely to tranquillize these alarming symptoms. I passed my hand into the uterus; it contained a good deal, I guess a pound and a half, of coagulated blood; I scooped it out with the hollow of my hand. There was no occasion to irritate the uterus to contract; the instant it was empty it shut like a spring, and when I put my hand on the outside I found it not more than half its previous size, but the symptoms continued as alarming as ever. I now gave her a dessert spoonful of Hoffman's æther, and fifty drops of laudanum, and then sent off for the family physician. When he arrived the symptoms had abated so much, as to relieve me from my anxiety; the pulse was slower, the breathing more tranquil, and she felt disposed to sleep. She continued to sleep nearly two hours while we remained in the house, then awoke for a few minutes, slept well through the night, and awoke the next morning without any vestige of her symptoms, sensible to herself; but her pulse continued frequent for many days." 156.

In illustration of the 2d position, (extraordinary momentum,) the following case is related. On the 10th of April, 1816, Dr. Gooch delivered Mrs. S. of her second child. For many hours before the accession of labour, she was flushed, and had a quick pulse. This state was diminished, but not removed, by proper means. It continued for some time *post partum*. After the removal of the placenta, the uterus felt, in the hypogastrium, contracted in the ordinary degree; yet, in twenty minutes afterwards, there came on one of the most frightful hæmorrhages he ever witnessed. By the introduction of the hand, and the application of cold, it was speedily arrested.

More than a year afterwards, the same lady came to town to be confined, and Dr. G. did not see her till she was in labour. On entering the chamber, he was struck with the same state of the circulation that had preceded her former accouchement—"she was sitting in her easy chair, with a red face and a throbbing pulse." In a few minutes the pains increased and it was necessary to put her to bed. The child was soon expelled, but gradually. The uterus contracted in the usual degree, yet, in a few minutes afterwards, the blood burst out with prodigious impetuosity, and gave rise to a fearful scene. The introduction of the hand and the application of cold speedily arrested the hæmorrhage. For many days afterwards, she could not sit up without faintness.

Our author wisely considered, that if she again became pregnant, it would be prudent to take such measures, before her accouchement, as might cause her to fall in labour with a cool skin and a quiet pulse. In twelve months afterwards, she informed our author, that she was five months gone with child. He advised her to avoid fermented liquors—to take meat only thrice a week—a purgative of salts and senna twice a week—a scruple of nitre thrice a day. This plan was to be begun two months before confinement. Our author saw her four days before her accouchement, and was gratified to find her with a cool skin and quiet pulse. Yet the former inflammatory state recurred, the same as before, for forty-eight hours before labour. The child was gradually expelled—the uterus contracted—but the hæmorrhage came on in a few minutes. It was, however, trifling, compared with the former attacks, and was easily suppressed by wetted napkins to the belly. In process of time, she again became pregnant, and to the precautionary means above described, she was twice moderately blooded, a little before confinement. Her labour, this time, was unaccompanied by flushing or quickened circulation, and no uterine hæmorrhage ensued.

Our experienced and intelligent author has met with other instances, similar in kind, though less striking in detail. He has seen a strong cordial, given unnecessarily towards the conclusion of a labour, excite inordinate action in the heart and arteries, the consequence of which was a flooding after the separation of the placenta, though the uterus was contracted in the ordinary degree. On the other hand, he has ventured to separate the placenta, while the uterus remained largely dilated; but the circulation being languid, no more blood was lost than after an ordinary labour.

"How often a disturbance of circulation plays an important part in uterine hæmorrhage it is difficult for an individual to know; but I suspect sufficiently often to deserve the especial attention of practitioners. I advise them when they meet with patients subject to hæmorrhage after delivery, to notice the state of the circulation before labour, and, if disturbed, to employ means for tranquillizing it before labour comes on. I advise them during labour, to use cordials cautiously, lest the placenta should separate during an excited state of circulation. I advise them after delivery, though the uterus may feel contracted, to be slow to leave their patient, if the circulation be greatly disturbed." 161.

Dr. Gooch alludes to the important subject of a contracted uterus becoming relaxed, and thus giving rise to hæmorrhage. The states of contraction and relaxation may, and do alternate, with corresponding cessations and recurrences of hæmorrhage. Thus, the first time he attended the lady, whose case has just been related, the bleeding returned again and again, although the abdomen had been covered with pounded ice—the uterus, at one time firm and distinct, becoming at another, so soft as no longer to be felt.

"Finding the ice so inefficient I swept it off, and taking an ewer of cold water, I let its contents fall from a height of several feet upon the belly; the effect was instantaneous; the uterus, which the moment before had been so soft and indistinct as not to be felt within the abdomen, became small and hard, the bleeding stopped and the faintness ceased; a striking proof of the important principle, that cold applied with a shock, is a more powerful means of producing contraction of the uterus, than a greater degree of cold without the shock." 163.

After the second labour, and at the beginning of the hæmorrhage, Dr. G. found the placenta separated and lying in the vagina. He removed it—the hæmorrhage abated, but returned in a few minutes. He took several handkerchiefs soaked in vinegar, and passed them, one after the other, into the vagina, so as completely to plug it. This prevented all external hæmorrage, and some uterine pains came on. But presently the pains ceased, the uterus softened and enlarged, and she turned ghastly pale. It was now evident, that an external, had been merely converted into an internal, hæmorrhage. He withdrew the handkerchiefs, and applied his hands in the manner described in the following extract, with perfect success.

"My belief now is, that when hæmorrhage occurs after the removal of the placenta, the quickest way to stop it, is to introduce the left hand closed within the uterus, apply the right hand open to the outside of the abdomen, and then between the two to compress the part where the placenta was attached, and from which chiefly the blood is flowing. When the hand is introduced merely as a stimulant, there is an interval of time between its arrival within the uterus and the secure contraction of this organ, during which much blood is often lost. By directing the hand to the very vessels from which it issues, and compressing them as I have described, this quantity is saved. If I may judge by my feeling, the blood stops, in a great degree, even before the uterus contracts; the hand acts first as a tourniquet, then as a stimulant. It is true we cannot tell with certainty where the placenta was attached, and consequently where the pressure should be applied; but as it is generally attached to or near the fundus, if the pressure be directed there, it will generally be right.

Besides, after the child is born, it is often several minutes before the placenta separates and descends; if, during this interval, we pass up the finger along the chord and observe at its entrance into the uterus, whether it turn towards the front, the back, the right or left side, or straight up to the fundus, we shall form a tolerably exact idea of the spot to which the placenta has been attached in this individual case." 165.

Dr. Good does not consider it necessary during syncope, in such cases, to delay introducing the hand until the patient revives. On the contrary, he introduces the hand, and endeavours to stop the blood, and induce contraction, before giving cordials or stimulants internally.

We have now given our readers a full and complete account of this valuable and truly practical paper. It is a great pity that Dr. Gooch does not write *more*—for very few can write *better*.

---

11. *Iritis.** Dr. Smith, of the Army Ophthalmic Depôt at Chatham, has made a valuable report of cases to our respected cotemporary of the North. Twelve instances of iritis are narrated, accompanied by the usual symptoms of increased vascularity of the iris and external tunics of the eye-ball, pain, intolerance of light, lachrymation, immobility, contraction, or irregularity of the pupil, dimness of vision, and sense of over-distention of the eye-ball. The cause was, in almost all the cases, attributed to the action of cold. Some of the men had had syphilis, and some not. The same might be said of mercury. These results, as far as they go, in Dr. Smith's opinion, "have little tendency to support the supposed noxious effects of syphilis or mercury on the eyes." Dr. Smith, from much experience, is inclined to view the syphilitic and mercurial actions as predisposing, not exciting causes of iritic inflammation, partly by their deranging the healthy functions of the system, and partly from their requiring confinement, whereby the body is rendered more susceptible to the action of cold, which appears to be the general exciting cause of idiopathic iritis.

The treatment was regulated by the severity of the symptoms. When the pain was violent, or moderate but of long standing, venesection to syncope was ordered—but when the pain was pretty moderate, and the other symptoms did not run high, local blood-letting was considered sufficient. After the bleeding, a purgative, generally containing some tartrite of antimony, was exhibited. No benefit was derived from full vomiting.

"As soon as the bowels were freely evacuated, the next object was to affect the mouth with mercury. This was done by giving calomel in conjunction with opium, during both the day and night,

---

* Dr. Smith. Ed. Journal, No. 73.

in doses of two grains of the former, and a quarter of a grain of the latter, every hour, or perhaps every two hours, till the gums felt tender, or an increased flow of saliva was manifested. About this period of the cure the patients generally stated that they experienced a very considerable abatement of the pain, and sense of fulness with which they had been hitherto annoyed. The vision immediately became more clear, and the irregularity of the pupil and effused lymph began to subside, and the iris to assume its natural colour. A continuation of the mercury for ten days or a fortnight, so as gently to keep the mouth affected, removed the disease in all except one."

Our author observes that iritis may be cured in the ordinary way, by antiphlogistics, but neither so safely nor so speedily as on the mercurial plan above described. Added to which, the chance of losing or impairing the visual orb is much more by the one treatment than by the other.

We have often been surprized that the treatment of iritis alone, which no one will deny to be an acute inflammation, did not alter the language of physicians and physiologists respecting the *stimulant* powers of mercury. It is considered a stimulus, a general or universal stimulus, and little else. Now wine or brandy is also a general stimulus; but would either of them cure iritis? The term (as a single one) most applicable to mercury is that of a *universal secernant;* and we have long thought that the increase of absorption consequent on the operation of mercury, results from the previous evacuation or depletion, which is well known, as in the instance of blood-letting, to set the absorbents actively at work in all parts of the system.

---

12. *Pectoral Disease—Hypochondrias—Suicide.** M. P. a celebrated gun-smith of Paris, 42 years of age, had enjoyed good health till the age of 23, when he experienced a pulmonic attack which left a chronic cough and expectoration of long standing. At this time he was attended by a friendly physician, who used to enter into long dissertations on the complaint and economy of the chest, &c. which greatly raised the patient's opinion of the doctor's skill. The latter promised a complete cure in six years; but at the end of three years put an end to his own existence without fulfilling his promise to the patient. The treatment during this first epoch consisted in general and local bleeding, and a caustic issue in the right leg to carry off the superabundance of humours which flowed to the lungs, and was discharged by coughing. In great despair at the death of his favourite physician, he determined to manage his health himself, but formed a project of acquiring a large fortune, in

---

* Retrecissement du coté droit de la Poitrine—Hypochondrie et Suicide. M. Beulac, M. D. Journal Complémentaire, No. 50.

the mean while, by perfecting all branches of his business. Before his wishes were quite accomplished he became so seriously ill, by intense application and great irregularity, that his wife insisted on his having medical assistance. At this epoch he exhibited symptoms of hydrothorax, as irregular chills, difficulty of breathing, sense of weight in the right side of the chest, and bulging out of the ribs of that side, to which was added, slow hectic fever. The celebrated Bayle now attended him. One night he suddenly discharged a large quantity of sero-purulent matter by coughing, after which the enlargement of the side became reduced, and, finally, that side of the thorax contracted. Cough and purulent expectoration continued. Being now in a state of demi-convalescence, M. P. went into the country, by the advice of his physicians, where he began to meditate profoundly on the " miseries of human life," and, for the first time, evinced an aversion to some of his nearest relations, especially his wife. The chimerical fear which he entertained of assassination induced him to always keep loaded pistols about his bed—and from this time may be dated his intellectual aberration.* In this state he continued, sometimes better and sometimes worse, for some years, viz. up to 1819. The treatment, during this epoch, consisted in expectorant ptisans, ".cinchona to check the hectic fever" —and a cautery to the right thigh. In 1819 there were some consultations between Drs. Martin, Cayol, Recamier, Louis-Villermay, M. Roux, and M. Laennec.† The right side of the thorax was found to be flattened and contracted—the shoulder depressed—the muscles wasted—the vertebral column slightly bent. When the stethoscope was applied, the breathing could be very faintly heard on this side, but very distinctly on the other. Pectoriloquism was evident between the clavicle and the edge of the trapezius muscle on this side, and intonation of the voice in the armpit. Two cauteries were applied to the vertebral column, and the sores kept open. During this period the patient manifestly began to suspect the fidelity of his wife—to look upon his affairs as in a very deranged condition ; and, in short, to exhibit the most unequivocal phenomena of mental alienation. In this condition he chose a lodging in the *Rue d'Enfer*—an ominous name for his abode—and determined not to go out of doors on any occasion. In a consultation of the forementioned physicians, it was determined to suppress the cauteries, and in their stead to keep open a blister. This measure, our author thinks, was the cause of the fatal catastrophe which followed—in which opinion we decidedly disagree with him. The patient had long entertained a notion that a person, in whom

---

* We do not quite agree with M. Beullac on this point. We think his mind was wavering before this period, and from the moment that he placed pistols in his bed-chamber to guard against assassination, his insanity was no longer a matter of doubt.

† This appears to be the case alluded to in the second volume of Laennec's work, page 371. *Rev.*

a blister failed to act, had but a short time to live. The blister did not act, and the patient concluded that his only method was now to commit suicide, to prevent the misery of a lingering death. He therefore put himself to death with one of the loaded pistols which hung at his bed-head!

Before making any reflections, we shall present the *post-mortem* appearances. The head was not opened—a most culpable omission under such circumstances. The right lung was nearly hepatized, and in its upper portion was found an excavation, capable of containing a very large nut, and communicating with several of the bronchia. There were false membranes in several places on this side glueing the lung to the diaphragm and ribs. The left lung was large and very sound, as were the heart and also the abdominal organs.

We may remark, in the first place, that nothing could be so highly improper as to allow a man who had evinced unquestionable symptoms of mental derangement for some years, or, indeed, for any time, to keep loaded pistols at his side, day and night. In the second place, it appears that the "pectoriloquie" and the "retrecissement du coté" absorbed the whole attention of the physicians, and that nothing was done to remove the cerebral malady. In the third place, the pulmonic affection was not of such a nature as to threaten life for some years to come; and therefore had the mental disease been removed, there was a prospect of the patients's enjoying life and the society of his friends for an indefinite period. The case affords a memorable lesson to the medical practitioner, never to permit a patient (as far as he can prevent it) who has aberration of intellect, to be master of his own actions for a single day.

---

13. *Compound Fractures.** The general practitioners in the country are treading close on the heels of the exclusive surgeons in town. There is a far greater spread now than formerly of medical and surgical information among all ranks of the profession, and they are become bolder and more successful in their practice. The pages of our own Journal have contained ample proofs of this—and the Medico-Chirurgical Transactions owe no small proportion of their contents to this valuable class of medical society.

Mr. Dunn is favourably known to the profession by a paper in a late volume of these transactions, containing a case of the removal of several of the tarsal and a part of the metatarsal bones followed by complete success. The present communication records two cases of compound fracture, in which the limbs have been saved by a similar removal of large portions from the middle of the cylindrical bones—and one of simple fracture, in which a projecting portion of

---

* Observations on Compound Fractures. By John Dunn, Esq. Surgeon, Scarborough. Med. Chir. Transactions, Vol. xii.

bone was sawn off with equal success. Of these cases we shall present our readers with a succinct analysis.

On the 17th of March, 1821, John Harper, a lad of 14 years of age, was thrown from his horse, and while one foot hung in the stirrup, the horse went off at full gallop. The consequence was a dreadful fracture of the right leg—"the broken ends of bone projecting from a wound of immense extent, and a portion of the tibia detached, which he (Mr. Hagyard) removed." Mr. Travis and Mr. Dunn arrived by candle-light—

"And found the poor lad in a small and wretched hovel, extended on a couch, with a large wound, and destruction of the skin of the middle of the leg; the upper portion of the tibia projecting like a stick, unconnected with any of the soft parts, and deprived even of its periosteum, to the extent of between two and three inches, and the lower portion denuded of all covering to the length of three-fourths of an inch. The fibula was also fractured near the knee, and in the centre of the leg, so that it was divided into three pieces. It was, however, so connected with the surrounding parts, that the spiculæ of bone could only be discovered by the insertion of the finger into the wound. The teguments on the posterior part of the limb, although much bruised, were not deadened; and the circulation could be distinctly traced along the course of the posterior tibial artery. A considerable hæmorrhage took place at the moment of the accident, but it was now suppressed. The wound was six inches or more in length, and as many in breadth; but the boy was comparatively tranquil. On consultation, the grand question was, whether to amputate the whole member; to put it up in splints as it was; or to saw off the denuded rough extremities of the tibia, and treat it as an ordinary compound fracture. In this dilemma, which required immediate decision, we determined upon the last expedient. The tourniquet was therefore applied, the broken ends of the bone raised from the wound, and whilst the limb was held steady by one, and a bone knife kept under the exposed portion of the tibia by another of my friends, I successfully amputated the two extremities of the fracture, including about *three* inches of the whole cylinder of the tibia. We were unable to reach the fibula with any instrument, so that the two portions of the tibia could not be brought within an inch and a half or two inches of each other, without projecting the spiculæ of the former into the surrounding muscles." 169.

Stitches were passed through the edges of the wound and their sides drawn as near as convenient, when strips of adhesive plaster were applied round the limb, with an eighteen tailed bandage and splints. The patient went on favourably—the wound became so covered with granulations that the bones were no longer discoverable. On the 1st of May the report was—"the sinuses diminished; the space between the bones filled up with solid matter; by compressing it on each side I could trace a continued line of bone." By the 26th of October the boy was walking about the streets.

While we give Mr. Dunn every credit for his judgment and prompt resolution, in the above case, and while we most fully approve of the practice which he adopted, we venture to differ from him on certain physiological points—and this difference, we have stated in our review of Sir Astley Cooper's work on Dislocations; we need not, therefore, repeat it here.—*See page* 633.

The second case related by Mr. Dunn, is that of a lad, sixteen years of age, who was stricken, on the middle of his leg, by a great plank of wood. Both the tibia and fibula were fractured. The ends of the former protruded from a very long wound, above half the length of his leg, having the appearance of a clean cut. The ends of the bones were very ragged—the most forcible extension could not place them in coaptation. It was, therefore, determined to saw off their extremities with the common amputating saw—No hæmorrhage of consequence followed—the wound was cleaned, and the bones put in apposition. About half an inch of the exterior part of the tibia was left denuded of its periosteum, but the rest of its circumference was connected with living parts. The leg was properly dressed, and the boy was judiciously treated. In four or five months the patient could walk without crutches.

The third case was the removal of a projecting edge of the tibia, after a badly united former fracture. These cases are very creditable, as we before observed, to Mr. Dunn, and to provincial surgery in general.

14. *Scrophula.** Mr. Fosbroke, whose zeal and active spirit of observation bid fair to promote the advancement of his profession, has recorded a curious example of the extent to which a local disease, depending upon a scrophulous diathesis, may go. The patient was a young man, 20 years of age, who had been attacked for years previously, with an inflammatory swelling of the left knee, which extended down to the foot. The swelling of the knee suppurated, and, by all accounts, discharged a great quantity of pus. The thigh then became suddenly swoln—the hip-joint became obviously affected—large and deep abscesses formed at the posterior part of the thigh, at the superior spinous processes of the ilium, on the sacrum and lumbar vertebræ, about the shoulder joint, under the true ribs, in each lumbar region, &c. sometimes discharging thick pus, sometimes thin matter, in great quantities. The right extremity was gradually enlarging like the left. In the latter, large portions of the tibia and femur had exfoliated, and abscesses had formed where the process of ulceration had afforded a passage for the removal of cylindrical portions of bone. Some portions were necrotic, and passed away covered with a bloody sanies. The cellular membrane and cutis were thickened, " and effusions of the cuticle, in large brown crusts, covered the surface of these limbs, now of extraordinary

* Mr. Fosbroke. Ed. Journal, No. 73.

magnitude." "These scaly crusts, with the general dense enlargement of the integuments, gave some resemblance to the horny hide of a rhinoceros." All contour was destroyed, and the size and deformity of the left leg exceeded that which is seen in elephantiasis. At the time Mr. Fosbroke saw the patient, all acute symptoms had subsided, and notwithstanding the dreadful condition he was in, the animal functions contended with great vigour against the progress of diseased action. "It is singular," says Mr. Fosbroke, "that the most vital parts exposed had so completely escaped, viz. the muscles, arteries, lymphatics, and the sheath of the medulla spinalis." On this account, probably, there was less waste of life than in common psoas abscess.

"My enquiries," says Mr. Fosbroke, "enable me to say, that scrophulous affections are more abundant on the southern coast than in more insular (we imagine this is a misprint for *inland)* situations. This fact, which is conformable to the opinions of Sir Astley Cooper, in his invaluable lectures, argues for the reverse of local representations."

15. *Suicide, curious tendency to.** A woman, 35 years of age, is now under the care of M. Falret for symptoms of phthisis. When 19 years old, the death of an uncle by his own hands, made a deep impression on her mind. She heard that insanity was hereditary, and the idea pursued her that she should one day fall into this wretched condition. She confessed her apprehensions only to the priest, who endeavoured to dissipate the mournful impression; and, in this state, she continued for two years, when the death of her reputed father, also, by suicide, riveted the conviction on her mind that her own doom was sealed. She was convinced that *her blood was corrupted*, and this idea appeared to her confirmed by her next menstrual secretion being less copious, and less coloured than usual. Tortured by this notion, she took the resolution of drowning herself. After leaving a letter in her chamber, apprising her friends of the manner of her meditated death, she plunged into the river, but being immediately drawn forth from the water, she was restored to life. The night following this attempt, she was harrassed with pain in the head; and, after a short sleep, she awoke incapable of recognizing any of the friends about her. She was evidently delirious, but made no allusion to her former melancholy apprehension. Although formerly religious and well-behaved, she now uttered nothing but obscenities. This delirious excitement continued three days, and was succeeded by melancholy and inclination to suicide. Headach again came on, with nausea and bilious vomitings, which, however, soon subsided. She emaciated considerably after this, and menstruation became irregular, being about once in three weeks, and scanty.

* M. Falret. Revue Medicale, December, 1821.

She was the picture of despair, and could not look at herself in a glass without terror. Once more she invoked the aid of religion, which afforded her some consolation, but was insufficient to dissipate her sufferings entirely. Meanwhile, her mother revealed to her the secret, that her real father was yet alive, and after sometime passed in scepticism on this point, she consented to an interview with her father. The physical resemblance was so striking, that all doubt was instantly removed from her mind. From that moment, all idea of suicide vanished—her spirits and health became progressively re-established—and the menses only continued irregular for a few months. Fourteen years have now elapsed since the attempt at self-destruction. She is the mother of three children, and, during her married state, has been reduced to the greatest penury and distress. But she has never, since the period alluded to, entertained the remotest idea of suicide—on the contrary, she has proved an exemplary wife and affectionate parent, having the full possession of her intellectual faculties.

The above case offers a good illustration of the power of moral emotions in producing physical lesions. The impression occasioned by her uncle's unhallowed death, evidently deranged her intellectual faculties. A melancholy, or *chronic* delirium preceded the attempt at suicide, and a maniacal, or *acute* delirium followed it. The effect of moral emotions was not less conspicuous in the cure, than in the production of the hallucination and tendency to suicide.

One other reflection we shall indulge in here—the impolicy of ever putting in rigid execution against the corpse, the inhuman laws against the crime. The only possible excuse for offering indignities to the lifeless clay, is the hope of deterring the living from following the example of the dead. But the revolting sensations occasioned by this cruel act, and the publicity and record of the act itself, are well calculated to produce, in the minds of relatives and even of strangers, that condition which leads to the catastrophe so much dreaded!

---

16. *Inflammation and Retroversion of the Uterus.** Dr. Robertson, of Glasgow, has detailed two or three interesting cases, in the Edinburgh Journal, where pain and intumescence, or inflammation of the uterus, in the unimpregnated female, appeared to produce a kind of retroversion of the organ, accompanied by a state of great suffering. The first case, however, is rather equivocal, and some people may still look upon it, as Dr. Robertson did, at first, himself, to be in some way connected with utero-gestation, though evident discharge of a blighted fœtus could not be perceived. The second case is less exceptionable, and we shall give some account of it.

Dr. R. was called to a young girl, 19 years of age, and found her resting on her knees and elbows, weeping from pain. Ten weeks previously, she had been delivered of a still-born child, and had had

* Dr. John Robertson. Ed. Journal, No. 73.

a good recovery. Two days before the present visit, her calls to make water became very frequent, without any known cause. (Ever since her confinement, however, she acknowledged that she had made water more frequently than formerly.) She now felt a dull uneasy weight in the loins, with slight pain in both groins. Her pulse was quick, feeble, even tremulous—pain in the umbilical and hypogastric regions, apparently agonizing—urine and fæces lately evacuated—no menstruation since last confinement. Sixty drops of laudanum were ordered, followed by a dose of castor oil and calomel. This was productive of temporary relief and sleep; but next morning she was as bad as before—principally complaining of her pubes, back, and groins—of frequent micturition and inclination to stool, the latter accompanied by much bearing-down pains. On examining, *per vaginam*, Dr. R. found the uterus very low down, and enlarged, as nearly as he could guess, to about the bulk of one's fist, "the fundus being directed backwards into the hollow of the sacrum, while the mouth, which was tumid and much indurated, was felt facing the lower edge of the symphisis pubis." The whole body of the womb was so tender, that she screamed when the most trifling pressure was made on it. Her pulse was quick, small, and more feeble than hard. During the night, she had been attacked with frequent vomiting. Venesectio ad ℥viij. which produced fainting. Fomentations and opium were then used to allay the pain. Pills, composed of calomel and opium, were next administered, with the view of reducing the bulk of the uterus. Leeches were, also, applied to the vagina and labia pudendi. On the 4th day from the commencement of the pills, the gums became tender. The uterus now felt somewhat flaccid—the tenesmus and dysury, in two days more, had almost subsided. The pills were continued at proper intervals so as to keep up a trifling irritation on the mouth, and on the 16th day from the time our author first saw her, she was well—the uterus feeling light, buoyant, and of the natural size. The symptoms and mode of treatment, in this case, were nearly the same as in the other, which we shall not notice.

That the phenomena, in these instances, were to be attributed to a congested, or sub-acutely inflamed state of the uterus, we can scarcely doubt; and that this state of turgescence produced the malposition, or retroversion, as it is called, we think is not very improbable. These inflammatory affections of the uterus are more common than is generally imagined, and the antiphlogistic treatment, especially by local blood-letting, rest, horizontal position, semicupia, and abstinence, is not sufficiently attended to. A farrago of remedies, supposed to have some specific influence on the uterine system, are too often substituted by the routinist for this rational treatment.

---

17. "*Test for Arsenic.** Dr. Cooper, president of Columbia

* Dr. Cooper. Sulliman's Journal.

College, finds a solution of chromate of potash to be one of the best tests of arsenic. One drop is turned green by the fourth of a grain of arsenic, by two or three drops of Fowler's mineral solution, or any other arsenite of potash. The arsenious acid takes oxygen from the chromate, which is converted into green oxide. To exhibit the effect, take, he says, five watch glasses; put on one, two or three drops of a (watery) solution of white arsenic; on the second, as much arsenite of potash; on the third, one-fourth of a grain of white arsenic in the substance; on the fourth, two or three drops of solution of corrosive sublimate, either in water or alcohol; in the fifth, two or three drops of a solution of copper. Add to each three or four drops of solution of chromate of potash. In half an hour, a bright, clear grass-green colour will appear in numbers 1, 2, 3, unchangeable by ammonia; number 4 will instantly exhibit an orange precipitate; number 5, a green, which a drop of ammonia will instantly change to blue. Dr. Cooper, however, does not recommend that this test should be exclusively relied on, but merely that it should be used in conjunction with others, of which the most unequivocal is certainly the actual exhibition of arsenic in a metallic form.

18. *Remarkable Case of Hepato-pulmonic Disease.** The Marquis de T———, sixty-eight years of age, had enjoyed good health till the year 1794, when he underwent excessive fatigue during the emigration, and was, soon after, seized with violent pain in the right hypochondrium, with all the symptoms of acute inflammation. A tumour now appeared in that region, and fluctuation soon became evident. This tumour burst spontaneously, and discharged an enormous quantity of a greenish purulent liquor, together with a great number of biliary calculi. It was long before this abscess healed. In the course of the succeeding ten years, four similar abscesses formed in the same place, and all accompanied by a discharge of gall-stones. After this epoch the Marquis's breathing was never very free, and he felt a tightness about the region of the liver, which inclined him to stoop in that direction, and which he attributed to adhesions formed during the different inflammatory attacks. Extreme emaciation now took place, accompanied by a slight cough, a very deep tinge of the skin, and a *tout ensemble* indicative of suffering—yet his appetite was good, his sleep tranquil, and the general functions of the system very little deranged. During the next twelve years he was frequently threatened with a return of the hypochondriac abscesses, but they were always prevented by local bleedings, diluent regimen, and topical emollients. About six months before his death an excessive difficulty of swallowing came on, which was sometimes mitigated by opiate embrocations, leeches, blisters, &c. but always returned, and was soon afterwards accompanied by

* M. Nacquart. Journal Gen. de Med. Juillet 1822.

an habitual and copious expuition of a glairy fluid. There was also occasional expectoration, without cough, of thick and puriform substance. The dysphagia now became so great that swallowing was nearly annihilated, and when any thing was got down, it was at the expense of pain the most intolerable. The whole hypochondriac region was now exceedingly sensible; the pulse became frequent; the puriform expectoration and glairy expuition increased, and the emaciation arrived at the most extraordinary pitch, notwithstanding the difficulty of swallowing decreased towards the close of life. Diarrhœa now supervened, and he died a perfect skeleton in June last.

*Dissection.* Peritoneum sound. Mucous membrane of the stomach was of a violet colour, and that of the colon sprinkled with ulcerations, some of them of considerable size. The liver was not half the natural size, and firmly agglutinated to the diaphragm by a white fibrous substance, in some places nearly as firm as cartilage. It was firmly adherent to the pylorus and other adjacent parts by similar fibrous substance. There was no trace of gall-bladder; but in its place was a depression occupied by fibrous substance similar to that which glued the liver to the neighbouring parts. The ductus communis choledochus was perfect, and the structure of the liver itself did not appear diseased, but was nearly white in colour. The left lung was sound, as were also the heart and pleura. The right lung was converted into a mass of tubercles, among which were several excavations produced by suppuration, (foyers de suppuration et des cavernes,) the whole firmly adherent to the pleura, which was itself disorganized. The fauces, pharynx, and œsophagus, examined with the greatest care, presented not a single trace of disease. The pia mater spread over the hemispheres of the brain was covered with a transparent gelatinous layer, but the brain itself was sound.

The above case will furnish food for much reflection and some speculation. The state of the lung on one side does not at all surprize us, for it would really appear that *one* lung is a great deal more than is necessary to support life, and even a tolerable degree of health. We have seen so many instance where the functions of respiration and circulation were kept up by a few cubic inches of permeable lung that nothing hardly can astonish us on that head. The dysphagia is a puzzler. The scoffers at "inexplicable *sympathy*" will, we apprehend, have some difficulty in accounting for this distressing symptom in any other way. How far the destruction of the gall-bladder and diminution of the liver itself were connected with the extreme emaciation and other phenomena, we will not pretend to say; but we are disposed to think that the disease in one side of the chest was not likely to be the sole cause of this emaciation. That the unnatural colour of the skin was connected with the hepatic derangement, will, we think, not be questioned. The state of integrity in which the parenchymatous structure of the liver was found (although the colour was altered) would lead us to suppose that the series of abscesses in the right hypochondrium were confined to the gall-bladder itself, of which we have seen some instances, and of which several other instances are on record.

19. *Epilepsy.** In a well written paper on epilepsy, in which Dr. Shearman advocates the doctrine of nervous, rather than *vascular*, disturbance, as the first link in the causation of the disease, we find the following passage. "The medicine which, in my hands, has more frequently succeeded than any other in removing epilepsy, is the elutriated oxyd of tin, given in the dose of from ℈ij. to ʒj. to an adult, night and morning, for about four days, at the end of that time giving a purgative, and again resuming the medicine or not, according to its effects upon the system, or its apparent power over the disease. That it possesses powers different from, and superior to the other preparations of the same metal, I am fully convinced, and I think it deserves a trial by practitioners after they have been disappointed of success in the exhibition of other remedies." Knowing Dr. Shearman to be a man of correct judgment and strict candour, we offer the above passage to the notice of our brethren at large.

---

20. *Strictured Œsophagus.*† Dr. Kinglake's case of *real* stricture in the œsophagus exhibits a striking contrast to the *supposed* case of stricture in the same number of our respected cotemporary. The site of the obstruction is not stated; but it was three inches in extent, and of almost cartilaginous hardness. The poor man died of inanition. Quicksilver to the extent of one or two ounces was given twice or thrice a week, which passed the strictured part, and descended through the bowels "rather in an unctuous than in a globular or metallic form." No pain or inconvenience was experienced by this process.

Our readers will remember that a few numbers back we stated a remarkable case of volvulus, where no traces could be found of the quicksilver which we exhibited to the patient the day before death. The above observation of Dr. Kinglake's seems to be somewhat similar.

While we congratulate Dr. Kinglake on the great increase of perspicuity in his language of late, we cannot help observing that it is still too redundant. The substance of this case might have been communicated in one page, as well as in four—and, as Pat says, "we should have gained by the loss." Dr. Kinglake, however, is not singular in his powers of amplification.

---

21. *Extraction of a living Fœtus from a dead Mother.*‡ It but rarely falls to the lot of a surgeon to have an operation of this kind on hand.

---

* Dr. Shearman. Medical Repository for September, 1822.

† Dr. Kinglake. Med. and Phys. Journal, Sept. 1822.

‡ Mr. Green. Med. Chir. Trans. vol. xii.

On the 15th of April, 1820, a woman in the last month of pregnancy was run over by a stage-coach near the end of St. Thomas's Street, Southwark. She was immediately conveyed to St. Thomas's Hospital, and expired in twenty minutes after the accident. Mr. Green and Dr. Blundell, after a short consultation, agreed on the propriety of the Cæsarean section, which was performed in less than a quarter of an hour from the death of the mother. On extracting the child it exhibited no signs of life. The umbilical cord was tied and divided—a tracheal pipe introduced, and the lungs inflated. After fifteen minutes artificial respiration the child shewed symptoms of returning life. The infant was then immersed in warm water, but the pulse diminished in force and frequency, and the breathing became embarrassed. It was now dipped in cold water, without any marked effect. After a time the breathing became natural, and in 52 minutes the child opened its eyes. It was taken by the friends to a house in the neighbourhood, and put under the care of a wet nurse. On the visit next day it was found that little nutriment had been taken—that the child had not cried—and that its breathing was embarrassed. The infant lived but 34 hours after emancipation from the womb of its unfortunate mother. On opening the body of the latter, it was discovered that the liver was rent through its substance by the crush of the wheels of the coach, and much blood extravasated in the abdomen.

This case, as Mr. Green observes, affords a proof that a fœtus may be recovered, if promptly extracted from the uterus, when the mother has been killed by violence—and this too, under the unfavourable circumstances of death, accompanied by a profuse hæmorrhage. The case detailed is creditable to the zeal, humanity, and ability of the two distinguished practitioners concerned.

---

22. *Puerperal Fever.** When Dr. Brennen proposed and exhibited so drastic a purgative as oil of turpentine in puerperal fever, it was considered preposterous, and our continental brethren still quote the circumstance as a fine example of British temerity in the exhibition of what they term heroic remedies. The medicine alluded to is now creeping into use in a great variety of complaints, and among others puerperal fever.

Dr. Payne exhibits his experience in rather vague facts. Most of them are from memory, and all have happened at more or less remote periods. He declaims against blood-letting—trembles for the consequences that are likely to happen from the use of the lancet—" agrees that the disease in question is of an inflammatory nature"—but believes that parturient women are less able to bear the loss of blood than under other circumstances. This is a rambling and inconsistent kind of pathology, which we do not much

---

* Dr. Henry Payne, of Nottingham. Ed. Journal, No. 73.

admire. If parturient women are less able to bear depletion—so, we should imagine, are they less able to bear the inflammatory disease for which it is used. When inflammation therefore exists in such cases, the sooner it is reduced the better. We strongly suspect that it is with blood-letting in inflammation, as with mercury in syphilis—many remedies will be proposed as substitutes before one is found to stand the competition. Dr. Payne states that oil of turpentine has cured every case of puerperal fever that has occurred to him during the last seven years! If Dr. Payne's experience has been considerable, the remedy, we may safely aver, is here overrated—if trifling, the success of the remedy should not have been stated in such positive terms. We are friendly to the medicine in question; but such sanguine friends as Dr. Payne would ruin any remedy, whatever was its merit.

---

23. *Purpura Hæmorrhagica.** Here we have oil of turpentine again. We thought, some time since, that colchicum would have banished the lancet and broke up Apothecaries' Hall:—oil of turpentine now threatens to do the same. What will be the next Herculean remedy to grapple with all kinds of diseases?

Dr. Nicholl has exhibited the oil of turpentine in three cases of purpura hæmorrhagica. Two of these cases were published in the Medical Repository for July 1821, and "in each of these cases there was an absence of every appearance which could call for the employment of venesection; in neither of them was there any visceral congestion; but the cause of the affection seemed to be referable to general want of tone in the extreme vessels." This view led our author to the ol. terebinth. and the result was most satisfactory. The recent or new case was a child two years and a half old, who was brought to Dr. Nicholl, her skin, mouth, gums, tongue, &c. being sprinkled with small black spots, like flea bites. The child was pallid and languid. Half a drachm of the oil with some syrup of senna and water was ordered thrice a day from the first till the 11th of December. The spots gradually died away; but the child being weak, some decoction of bark and acid was prescribed. There was a return of the complaint in March. The terebinthinate and cinchonic medicines were ordered to be taken alternately, and they were persevered in till June, when the child appeared free from the disease. Our readers will readily grant that there is nothing very decided or satisfactory in this case, when we consider that other remedies were conjoined with the turpentine, and that the time occupied with the relapse afforded Nature an ample scope for the cure of the disease. At the same time we are disposed to think that the oil may be a serviceable medicine in certain conditions of purpura that do not indicate the use of the lancet.

---

* Dr. Whitlock Nicholl.

24. *Tartrite of Antimony in Pulmonic Inflammations.** Our professional brethren in this country have very generally been sceptical as to the large doses of certain powerful medicines, especially emetic tartar, exhibited by the Italian physicians. We have lately seen several English medical men, who were eye-witnesses to these administrations, and they confidently assert, *first*, that the medicines were of the usual strength, and *secondly*, that these large doses did not produce those effects which we would be led to expect from their comparative magnitude. We all remember that when the East India practitioners first prescribed scruple or half-drachm doses of calomel, the *facts* were denied by some in this country, because the *effects* were not what they hypothetically considered they *should* be. They are now less incredulous on this subject, and therefore we should not be entirely sceptical as to the practice of our Italian brethren, who have eyes, ears, and brains, as well as ourselves.

The writer of the paper before us resides in the Canton de Vaud, where inflammatory affections of the chest are very frequent and severe. For several years past he has given up all sanguineous depletion and counter-irritation in the treatment of this class of complaints, and trusted entirely to large doses of emetic tartar. He exhibits, for instance, from six to twelve or fifteen grains in the 24 hours, dissolved in a six-ounce mixture, of which he gives a table-spoonful every two hours in abundance of a common laxative ptisan. If there occurred much tendency to perspiration, he added two drachms of the spir. ætheris nitrici—and where there was much uneasiness and insomnium, a drachm of tincture of opium was added to the mixture. In general the quantity of emetic tartar was increased three grains daily till the patient took twelve or fifteen grains per diem—a quantum which he has rarely had occasion to exceed. The following were the usual effects of the medicine. The patients generally vomited after the second or third dose of the first mixture, and afterwards it either acted on the bowels, or produced no other sensible effect than that of mitigating quickly the symptoms of the disease. The patients generally expressed themselves as very much soothed about the chest, and when the usual doses were delayed, they complained of not being so well. "I have to remark," says our author, "that large doses of the tartrite produced much less vomiting than small doses which always gave rise to great distress without beneficial results." In the greater number of cases this mode of treatment did not require more than eight days—it was rarely prolonged to a fortnight. In a few cases a blister was applied to the painful part, but neither local nor general bleeding was ever used. No case of pulmonic inflammation terminated fatally in our author's hands since he commenced this method of treatment.

* M. Peschier. Revue Med. Aug. 1822.

25. *Cystitis.** The unfortunate subject of this paper fell a victim to his curiosity. Having ascended an unfinished scaffold to get a view of the pageantry attending the regalia in its removal from the Castle to Holyrood, (12th August, 1822,) he and several others were wounded by the erection giving way. He had some ribs broken, and a bruise on the loins, which deprived him partially of the power of the lower extremities. Being carried to the Royal Infirmary, he was there cupped and scarified, but, according to his own account, without effect. There being retention of urine, the catheter was introduced in the evening without difficulty, but during the two succeeding days and nights, the patient reported that the efforts of the surgeons were ineffectual in drawing off the water. On the third morning his friends removed him from the infirmary, and on the fourth day Mr. Liston was sent for, who found the bladder reaching to the umbilicus. A full sized catheter was introduced without any difficulty, and two pints of putrid fluid, more resembling blood than urine, were discharged. The fluid was drawn off every day once or twice, for some days, and hopes were entertained of his recovery. But these hopes were fallacious. On the 2d of September, the catheter was passed, but failed to draw off any fluid, and in the evening the symptoms were so threatening that Mr. Liston was obliged to take some more active steps. The bladder could be felt as a circumscribed doughy tumour rising into the abdomen, the superincumbent integuments being œdematous. The catheter was again passed with ease, but only a small quantity of offensive pus came away. The bladder was therefore opened freely by striking a sharp-pointed bistoury into the anterior part above the pubes, and thus making an opening sufficient to admit the finger. A great quantity of putrid purulent matter came away, but was stopped by the protrusion of a *flocculent membrane.* This was withdrawn by the fingers, and the bladder completely emptied. The lining of the bladder was found to be rough, and had lost the disposition to contract. The patient, from being apparently moribund, was greatly relieved; but at his age success could hardly be expected. He lived till the 20th of the same month. The separated membrane was apparently the product of recent inflammatory action; but whether from over-distention of the organ or other cause we feel incapable of saying. We think Mr. Liston was perfectly justifiable in performing the operation above-mentioned under the desperate circumstances in which the poor man was placed, and we wish that able and zealous surgeon all possible success in his professional career.

---

26. *Elongated Uvula*†. Dr. Physic and the editors of the Philadelphia Journal of Medical Sciences desire to draw the attention of their professional brethren to a species of consumption, in many instances of a very formidable character, produced by the irritation

---

* Mr. Liston. Med. Repos. No. 107. Nov. 1822.

† Dr. Chapman's Journal, No. 8.

of an elongated uvula, and which is relieved by simply cutting off a portion of it. Whether such a circumstance might give rise to disease of the larynx we cannot say, but we have seen three instances, within the last twelve months, where the relaxed uvula, by keeping up a constant tickling cough, rendered the patients very uncomfortable—indeed very unwell. In two of the cases, the complaint was subdued, though tediously, by stiptic and stimulating gargles—in the other case the tip of the uvula was snipped off by a pair of scissars, and the patient was quickly cured.

In the same journal we are informed that the superacetite of lead combined with opium is pretty freely exhibited in America for several affections of the bowels—especially the cholera infantum and dysentery. To an adult they give from half a grain to a grain of the lead with five drops of tincture of opium every hour or two, according to the urgency of the symptoms. In some instances, where it was desirable to act upon the skin, small doses of ipecacuan were added. Occasionally the medicine was omitted, and purgatives exhibited.

---

27. *Experiments upon the Roots of the Spinal Nerves.** M. Magendie had been, for some time, anxious to make an experiment for the purpose of observing the effects of the section of the posterior roots of the nerves which have their origin in the spinal marrow. He had attempted it several times, but without being able to succeed, in consequence of the difficulty of opening the vertebral canal, without injuring the spinal marrow, and producing death, or, "at the least, severely wounding the animal." During the last month, however, a litter of puppies having been brought into his laboratory, he considered them proper subjects for a repetition of his experiment in opening the vertebral canal. With a single stroke of a very sharp scalpel he laid bare the posterior half of the spinal marrow, covered with its envelopes. He then divided, with ease, the dura mater surrounding it, and exposed the posterior roots of the lumbar and sacral pairs, and, by raising them with the blades of a pair of small scissars, he was enabled to cut them on one side, without injuring the spinal marrow: the wound was reunited by suture, and the animal watched. M. Magendie at first believed that the limb corresponding to the divided nerves was entirely paralyzed: it was insensible to punctures and the strongest pressure, and seemed likewise immoveable; but, to his great surprize, the animal began to move it, in a very apparent manner, although the sensibility was still quite extinct. A second and third experiment gave an exactly similar result. M. Magendie then began "to consider it probable that the posterior roots of the spinal nerves might have different functions from the anterior, and that they were more particularly destined for sensibility."

He then thought of dividing the anterior roots, leaving the posterior untouched; but he found this easier to conceive than to exe-

* Magendie's Physiological Journal.

cute. After some consideration, he decided upon attempting to pass before the posterior roots a species of cataract knife, the blade of which being very narrow, might permit him to cut the roots by pressing them with the edge of the instrument, upon the posterior part of the bodies of the vertebræ; but this plan he was obliged to quit, in consequence of the great veins which the canal on that side contained, and which were opened at each movement in advance. In making these attempts, he perceived that towards the *vertebral dura mater* the anterior roots might be seen united into fasciæ, immediately before piercing that membrane, M. Magendie immediately divided all the pairs which he was desirous of doing, but, as in the preceding experiments, only cut those of one side, for the purpose of comparison: the limb became immediately lax and immoveable, whilst it unequivocally preserved its sensibility. He finally cut, at once, both the anterior and posterior branches, when there was an absolute loss of both sensibility and motion. "I have repeated and varied," says M. Magendie, "these experiments in several species of animals: the results which I have just mentioned have been confirmed in the most complete manner, both as regards the anterior and posterior roots. I am following up these researches, and shall give a more detailed account of them in the next number; it is enough for me, at present, to be enabled to assert positively, that the anterior and posterior roots of the nerves which spring from the spinal marrow have different functions; that the posterior appear more particularly destined for sensibility, whilst the anterior seem more especially connected with motion."

28. *Apparatus for Removing Poisons from the Stomach, invented by Mr. Jukes and Mr. Bush.** It (Mr. Jukes's) consists of an elastic gum tube, a quarter of an inch in diameter, and two feet and a half in length, terminating at one extremity in a small globe of ivory, with several perforations; the other extremity is adapted, either by screw or by plug (the latter is preferable) to an elastic *bottle* of sufficient size to contain at least a quart of liquid, and having a stop-cock fitted to it, in a similar manner as in the hydrocele bottle. Instead of the *bottle*, a pewter *syringe*, of an equal capacity, may be adapted, in the same manner, to the flexible tube. The operation by the syringe is performed more quickly, and may therefore, perhaps, be preferred by some. In cases where surgeons have neither *bottle* nor *syringe*, the *tube alone* might be made to answer the purpose, if the operator apply his mouth to its extremity, and thereby institute the office of a siphon.

*Application.* The patient ought to be placed on the left side, and the *globulated* end of the tube be then carefully passed to the greater curvature of the stomach, either through the mouth or nostril, as

* Med. Repos. for October, 1822; London Med. and Phys. Journal, September, 1822.

may be thought proper. Having previously filled the *bottle* or *syringe* with warm water, at the temperature of 150°, screw or plug it to the tube, turn the stop-cock, and gently force the contents into the stomach. The then diluted contents are to be immediately withdrawn by pulling up the piston; or, if the *bottle* be applied, the same effect will ensue from its elasticity enabling it to recover its original form, by which the fluid contents will return, charged with the poison. This operation ought to be repeated, till the water, which is withdrawn, becomes clear and tasteless.

In Mr. Juke's experiments, first on dogs, and then on himself and others, assisted by Mr. James Scott, Surgeon, in Westminster, the apparatus was supposed fully to answer the intended purpose. In these experiments, Mr. Jukes swallowed, first, *two drams* of laudanum; he afterwards gradually increased the quantity, until it reached ten drams: since which, he has administered to several individuals (one of them a female) *one* ounce of laudanum with an equally successful result. If the experience of others should confirm the above statement, Mr. Jukes will have conferred an important benefit on the profession.

Mr. Bush, of Frome, has proposed a similar apparatus in the September number of the Medical and Physical Journal; but from the violent and unmannerly attack which has been made on that gentleman through the medium of a popular journal, the name of which we do not choose to introduce into our pages, but with which Mr. Jukes's friend is reported to be connected, we augur no good to the public—but only a job for filling the pockets of some individual. At the same time we have reason to believe that Mr. Jukes himself is a respectable and a well-informed practitioner.

29. *Attempt at Suicide by swallowing a Key.** The following case among many others will excite some melancholy reflections on the fate of our late minister for foreign affairs. On the 28th April last, M. Priorry was called to the hotel de la Bibliothèque, where he found a man of athletic form and military appearance, in a state of complete insensibility. He had gone to bed in apparent tranquillity the night before, and was found, at a late hour next morning, lying on the ground in the state above described. On examination, M. Priorry observed that the face was flushed, tumid, and the vessels injected—the lips lived—an ecchymosis on the left cheek—contused appearances on the neck—complete immobility—nausea, and vomiting of a frothy slime—tongue clean—respiration embarrassed—pulse frequent and strong—apparent abolition of sense—no answers to any questions.

M. Priorry naturally asked himself, what is the cause of all these phenomena? Has the patient experienced an attack of epilepsy?

* M. Priorry. Journ. Gen. de Medicine, Juillet 1822.

Has he attempted to strangle himself? Has he taken some poisonous substance? and, if so, what is the nature of that substance? These were questions which naturally occurred to the medical attendant, but were not easily resolved. Not knowing what to do, and in order to gain time, M. Priorry endeavoured to make the patient swallow some spoonfuls of sugar and water, (eau sucrée,) which were immediately vomited up. He next determined on opening a vein—a measure that was clearly indicated at all events—but just as he was preparing for the venesection, he observed that the patient opened a little his eyes. He reiterated his questions—M. B. lifted up, with difficulty, the right hand, and made a motion as if turning a key of a door. It instantly struck our author that the miserable man had swallowed a key—the mode in which the unfortunate Gilbert had perished, and on pushing his fingers into the pharynx, he was soon convinced that the key of the chamber door was lodged in the œsophagus! Professor Roux was now sent for, and, after several unsuccessful attempts, the key, together with an oblong piece of copper attached by a chain to the handle of the instrument, were extracted from the throat. The alarming symptoms immediately subsided; but the irritation and inflammation occasioned by the foreign body required prompt and decisive depletions, both local and general. Presently his speech was restored, but he refused to give any account of the motives which led to the suicidal attempt. In the succeeding night he made fresh efforts to destroy himself; first, by hanging with the bed-clothes—and that failing, he endeavoured to strangle himself by squeezing two chairs against his neck. These attempts proving insufficient, he again swallowed the same key as far as he could possibly push it down his throat! He was nearly dead when found in the morning; and now the course was pursued which ought to have been pursued in the beginning. He was taken to the hospital, the key extracted, a straight waistcoat applied, and rigid discipline, in respect to depletion and diet, enjoined. By these means all disposition to suicide, in other words, the mental alienation under which he laboured, was soon subdued, and he left the hospital in perfect integrity of mind.

This is a good example elucidating the necessity of guarding a person by the strictest surveillance from the moment that he evinces the slightest symptom of mental alienation—*whether the aberration manifests itself by incongruous expressions or attempts at self-destruction.* This precept should be engraven on the mind of every medical man, and no circumstance should prevent his unfolding it to the parties concerned the moment it is necessary. Procrastination is not, in these cases, merely the "thief of time," but actually the executioner of the unhappy patient.

---

30. *Spinal Distortion.** Mr. Bampfield, who has lately been directing a good deal of attention to the subject of spinal distortion,

* Mr. Bampfield. Med. and Phys. Journal, Nov. 1822.

is now publishing an essay on the complaint in our respected cotemporary. The general principle of cure proposed to be acted on by our author, is not to procure anchylosis by rest and the horizontal position, but to prevent that process by restoring the vertebral apparatus to its pristine integrity of structure and function. By this we believe he means that in the majority of cases where the spine deviates, especially in its earlier stages, from the natural line, there is *not* any ulcerative process that would (if it existed) render anchylosis desirable. What the nature of the diseased state in the bone is, we cannot learn from that part of the Essay yet published; but he seems convinced that it is not ulceration.

The modus medendi of our author differs from that of others, as far as we know, inasmuch as he confines his patients on their bellies instead of on their backs, and makes pressure on the projections (this is supposing the spine to project outwards, or, as he not inaptly terms the disease, "excurvation,") by means of a pad, shield, and long roller passed round the body. The apparatus is to be removed from time to time, and manual efforts used to press back the bones into their proper situation. Two pillows are placed under the belly of the patient, and the general health is, of course, to be attended to. The principal physiological reason for Mr. Bampfield's preference of the facial hosizontal position in excurvations of the spine appears to be the action of the psoas magnus muscle on the last dorsal and on the lumbar vertebræ; which action, he thinks, will necessarily draw those vertebræ inwards, (when the muscle is on the stretch in the facial position,) and thus strongly promote the cure. We leave it to time and further experience to ascertain the value of Mr. Bampfield's proposals.

---

31. *Limitation of Emetics.* Dr. Sutton, of Greenwich, has written a short paper in the November number of the Medical Repository, to shew that where we wish to limit the operation of emetics to the stomach, and prevent their action on the bowels, we should add five or six drops of laudanum to the emetic draught, which, in his experience, has answered the purpose in question.

---

32. *Gonorrhœa.** Mr. Churchill has made several judicious observations on the treatment of gonorrhœa—none of them very new excepting the following precept, which we shall here extract. "The company of women should be avoided, and all those various causes which excite sensual ideas; to secure which, we should discard feather beds and their warm appendages. To the students of Oxford and Cambridge I recommend attention to Euclid; to those of the law, a revisal of the statute book; while those of my own profession will do well to read Barclay on Muscular Motion, or Hutchinson on Infanticide."

---

* Mr. Churchill. Med. Repos. 104-5.

